# Lowering the increased intracellular pH of human‐induced pluripotent stem cell‐derived endothelial cells induces formation of mature Weibel‐Palade bodies

**DOI:** 10.1002/sctm.19-0392

**Published:** 2020-03-12

**Authors:** Gesa L. Tiemeier, Rozemarijn de Koning, Gangqi Wang, Sarantos Kostidis, Rosalie G. J. Rietjens, Wendy M. P. J. Sol, Sébastien J. Dumas, Martin Giera, Cathelijne W. van den Berg, Jeroen C. J. Eikenboom, Bernard M. van den Berg, Peter Carmeliet, Ton J. Rabelink

**Affiliations:** ^1^ The Einthoven Laboratory for Vascular and Regenerative Medicine, Division of Nephrology, Department of Internal Medicine Leiden University Medical Center Leiden The Netherlands; ^2^ Center for Proteomics and Metabolomics Leiden University Medical Center Leiden The Netherlands; ^3^ Laboratory of Angiogenesis and Vascular Metabolism, Department of Oncology, KU Leuven Leuven Belgium; ^4^ Laboratory of Angiogenesis and Vascular Metabolism Center for Cancer Biology, VIB Leuven Belgium; ^5^ The Einthoven Laboratory for Experimental Vascular Medicine, Department of Thrombosis and Hemostasis Leiden University Medical Center Leiden The Netherlands

**Keywords:** cell therapy, glycolysis, induced pluripotent stem cell‐derived endothelial cells, von Willebrand factor, Weibel‐Palade bodies

## Abstract

Differentiation of human‐induced pluripotent stem cells (hiPSCs) into vascular endothelium is of great importance to tissue engineering, disease modeling, and use in regenerative medicine. Although differentiation of hiPSCs into endothelial‐like cells (hiPSC‐derived endothelial cells [hiPSC‐ECs]) has been demonstrated before, controversy exists as to what extent these cells faithfully reflect mature endothelium. To address this issue, we investigate hiPSC‐ECs maturation by their ability to express von Willebrand factor (VWF) and formation of Weibel‐Palade bodies (WPBs). Using multiple hiPSCs lines, hiPSC‐ECs failed to form proper VWF and WPBs, essential for angiogenesis, primary and secondary homeostasis. Lowering the increased intracellular pH (pHi) of hiPSC‐ECs with acetic acid did result in the formation of elongated WPBs. Nuclear magnetic resonance data showed that the higher pHi in hiPSC‐ECs occurred in association with decreased intracellular lactate concentrations. This was explained by decreased glycolytic flux toward pyruvate and lactate in hiPSC‐ECs. In addition, decreased expression of monocarboxylate transporter member 1, a member of the solute carrier family (SLC16A1), which regulates lactate and H+ uptake, contributed to the high pHi of hiPSC‐EC. Mechanistically, pro‐VWF dimers require the lower pH environment of the *trans*‐Golgi network for maturation and tubulation. These data show that while hiPSC‐ECs may share many features with mature EC, they are characterized by metabolic immaturity hampering proper EC function.

## Graphical abstract

Formation of mature Weibel‐Palade bodies in human‐induced pluripotent stem cell‐derived endothelial cells is limited by the increased intracellular pH (pHi) around the Golgi apparatus and *trans*‐Golgi network. This increase in pHi is caused by reduced intracellular lactate accompanied by reduced H+, caused by reduced glycolysis, and reduced the uptake of lactate and H+ via monocarboxylate transporter member 1.

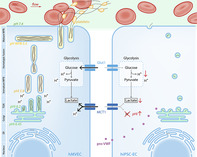

**Significance statement**

The formation of functional and mature Weibel‐Palade bodies (WPBs), essential for endothelial cell (EC) function, in human induced pluripotent stem cell (hiPSC)‐ECs is a crucial step in the development of the full potential of hiPSC‐EC for tissue regeneration, organ vascularization, and disease modeling. Current differentiation protocols fail to create hiPSC‐EC with mature WPBs in vitro. To the best of the authors' knowledge, this is the first study with detailed characterization of von Willebrand factor (VWF) and WPBs in hiPSC‐ECs and 13C‐labeled glucose flux metabolomics of hiPSC‐ECs. The results of this study show that hiPSC‐ECs have a higher intracellular pH (pHi) than mature EC, where pro‐VWF dimers require the lower pH environment for maturation and tubulation. Metabolic experiments showed that higher pHi in hiPSC‐ECs occurred in association with decreased glycolysis and reduced intracellular lactate concentrations. In addition, decreased expression of MCT1, a pHi‐sensitive member of the solute carrier family (SLC16A1), which regulates lactate and H+ uptake was observed. By lowering pHi with acetic acid, formation of mature WPBs in hiPSC‐ECs could be induced.

## 1 INTRODUCTION

Recent developments in stem cell biology have enabled differentiation of reprogrammed cells into the cell type of interest, a phenomenon that opened up many opportunities for studying organogenesis and repair, disease modeling with organoids, and patient‐specific cell therapy. A functional vasculature, however, is a prerequisite to realize these opportunities and differentiation of human‐induced pluripotent stem cells (hiPSCs) into proper functioning endothelium is a key to unlock the potential of reprogramming‐based strategies.^1^, ^2^, ^3^, ^4^, ^5^, ^6^, ^7^ Recent studies characterized hiPSC‐derived endothelial cells (hiPSC‐ECs) and concluded that although hiPSC‐ECs demonstrate a spectrum of physiological endothelial functions, the punctate perinuclear localization of von Willebrand factor (VWF) may suggest an immature stage of Weibel‐Palade bodies (WPBs) biogenesis.^8^, ^9^

Endothelial cells (ECs) are key regulators of vascular hemostasis by producing VWF, a multimeric glycoprotein.^10^, ^11^, ^12^ After excretion at the site of vascular injury, VWF chaperones and protects blood coagulation factor VIII from proteolytic inactivation and binds to glycoprotein Ib receptors on platelets, as well as collagen and heparin, and is therefore involved in both primary and secondary hemostases.^11^, ^13^ Biosynthesis of VWF emerges in the endoplasmic reticulum (ER), where VWF precursor proteins consisting of a signal peptide, N‐terminal propeptide, and a mature peptide start to dimerize through the C‐terminal cysteine knot (CK) domain interactions. Subsequently, disulfide bonding between D′ and D3 domains initiates multimerization in the Golgi.^14^, ^15^, ^16^, ^17^ The final processing of VWF takes place at the *trans*‐Golgi network (TGN) where pro‐VWF is cleaved by furin and VWF is either stored in endothelial‐specific elongated cigar‐shaped secretory organelles named WPBs or constitutively secreted.^14^, ^18^, ^19^, ^20^ Prior to WPB storage, highly multimerized VWF proteins start to form tubules, which create the striated appearance of WPBs. WPB formation is driven by the presence of VWF protein as introduction of VWF in non‐ECs has shown to stimulate formation of WPB‐like storage organelles.^18^, ^21^, ^22^ Tubulation allows a 100‐fold compaction of VWF and drives the formation of elongated WPBs, which is achieved through low‐pH‐dependent interactions between the VWF propeptide and N‐terminal region of mature VWF.^20^, ^23^ Furthermore, tubulation is necessary for the rapid unfurling of ultra‐long VWF filaments, caused by the shift from the acidic WPB (pH 5.5) to the neutral pH of plasma during exocytosis.^14^ In addition, tubulation and thus WPB elongation influence VWF function as shorter WPBs excrete VWF with a reduced functionality.^24^ Besides VWF, WPBs also store angiopoietin‐2 (Ang‐2), p‐selectin, interleukin‐8, eotaxin‐3, calcitonin gene‐related peptide, endothelin (EDN1), endothelin‐converting enzyme 1, CD63, alpha 1,3‐fucosyltransferase VI, tissue‐type plasminogen activator, osteoprotegerin, factor VIII, and Rab proteins,^11^, ^25^, ^26^, ^27^ indicating the importance of functional WPBs as all these molecules are essential for proper endothelial performance.^20^ The maturation process of VWF is an important determinant of recruitment and composition of WPB cargo into the TGN.^12^, ^28^

Shear stress is a key regulator of VWF gene expression, WPB formation, and stimulates ECs to switch from a migratory and proliferative state during angiogenesis (tip and stalk cells) to a quiescent state (phalanx cells).^24^, ^27^ Physiological shear stress also initiates a vasodilatory, antithrombotic, anti‐inflammatory, and antioxidant phenotype and therefore is atheroprotective.^26^ Blood flow induces upregulation of transcription factor Krüppel‐like factor 2 (KLF2), which has proven to consistently control the expression of many genes involved in functionally mature endothelium as it acts as a transcriptional switch between the quiescent and activated states.^24^, ^29^, ^30^ Another important regulatory environmental cue in formation and stabilization of the vasculature derives from pericytes, mural cells originating from the vascular smooth muscle lineage which line capillary walls.^31^ These cells are involved in the regulation of angiogenesis, blood flow, structural stabilization of the vasculature, and vascular permeability.^32^ Taken together, stimulation of KLF2 expression and coculture with pericytes are both involved in the stabilization of vasculature and could provide an instructive environment for hiPSC‐ECs maturation.

Here, we explored the effect of environmental stimuli as shear stress, KLF2 overexpression, and pericyte coculture on the VWF and WPB phenotype as a standard for hiPSC‐ECs maturity. As shear stress in EC is also a key regulator of glycolysis, we hypothesized that associated changes in cell metabolism in hiPSC‐ECs may be a modulator of VWF and WPB maturation.

## 2 MATERIALS AND METHODS

### 2.1 hiPSC culture and EC differentiation

NCRM1 was obtained from RUCDR Infinite Biologics (Piscataway, New Jersey; generated by reprogramming of CD34+ cord blood using episomal vectors). LUMC0072iCTRL01 (L72) and LUMC0099iCTRL04 (L99) were generated by the Leiden University Medical Center (LUMC) iPSC core facility on mouse embryonic fibroblasts using Simplicon RNA Reprogramming Kit (Millipore‐Merck, Amsterdam, the Netherlands) and ReproRNA (STEMCELL Technologies, Köln, Germany), respectively, as described previously^33^ and further cultured in TeSR‐E8 medium (STEMCELL Technologies). The hiPSC‐ECs were generated from these lines according to Orlova et al^3^ and after isolation, hiPSC‐ECs were transferred to EC serum‐free medium (EC‐SFM, Gibco, Thermo Fisher Scientific, Waltham, Massachusetts) to which platelet‐poor plasma serum (1% vol/vol) (Biomedical Technologies Inc., Stoughton, Massachusetts), 50 μg/mL vascularendothelial growth factor (VEGF)‐165 (R&D Systems, Biomedical Technologies) and 100 μg/mL basic fibroblast growth factor (Miltenyi Biotech, Bergisch Gladbach, Germany), VEGF and basic fibroblast growth factor had been added (EC‐SFM full medium) at 37°C with 5% CO_2_ and antibiotics (100 IU/mL penicillin and 100 μg/mL streptomycin) as described in detail previously.^34^ Results of RNA sequencing were obtained and reproduced in ECs from three different hiPSC lines (hiPSC‐L72, hiPSC‐L99, and hiPSC‐NCRM1). All other results are from NCRM1 (also used as undifferentiated control).

### 2.2 Primary human microvascular ECs culture

Human microvascular ECs (hMVECs) are isolated from human kidney cortical tissue and were purchased from Cell Systems (ACBRI‐128, Kirkland, Washington). They were available at Passage 3 (<12 cumulative population doublings) cryopreserved in choline‐substituted cryopreservation (CSC) cell‐freezing medium (4Z0‐705). hMVECs were also cultured in EC‐SFM full medium at 37°C with 5% CO_2_ and antibiotics (100 IU/mL penicillin and 100 μg/mL streptomycin).

### 2.3 Human kidney‐derived perivascular stromal cells culture

Human kidney‐derived perivascular stromal cells (hkPSCs) were isolated from transplant‐grade kidneys discarded for as surgical waste.^35^ fluorescence‐activated cell sorting (FACS) confirmed that homogeneous NG2‐positive hkPSC populations between passages 4 and 8 and hMVEC between passages 6 and 8 were cultured in EC‐SFM full medium at 37°C and 5% CO_2_ as described in detail previously.^34^

### 2.4 Flow experiments

Shear experiments were performed using an Ibidi flow system (Ibidi, Martinsried, Germany) and described elsewhere.^34^ In short, cells were cultured for 4 days at a constant laminar shear stress of 5 dyne/cm^2^ in EC‐SFM full medium. Cells were seeded into closed perfusion chambers (IbiTreat 0.4‐μ slide I or VI, Luer; Ibidi) coated with 0.2% porcine gelatin (Sigma‐Aldrich, St. Louis, Missouri) at a concentration of 1.5 × 106 cells/mL and allowed to adhere for 3 hours. Thereafter, the chamber was connected to a computer‐controlled air pressure pump and a fluidic unit with a two‐way switching valve. The pump setup allowed pumping of 16 mL of cell culture medium from two reservoirs in a unidirectional way through the flow channel over the monolayer of ECs at a constant shear stress of 5 dyne/cm^2^. Medium was refreshed after day 1 of culture. The chamber and the reservoirs containing the medium are kept in an incubator at 37°C and 5% CO_2_. RNA was isolated from cells subjected to shear stress in a 0.4‐μ slide I Luer flow chamber, whereas the six lanes of a 0.4‐μ slide VI Luer are used for immunofluorescent staining.

### 2.5 Confocal immunofluorescence microscopy

After exposure to flow or static conditions (at day 4), hMVECs and hiPSC‐ECs were fixed in freshly made 4% paraformaldehyde (Alfa Aesar, Thermo Fisher Scientific, Haverhill, Massachusetts) in Hanks' balanced saline solution (HBSS; Invitrogen, Thermo Fisher Scientific, Carslbad, California) for 10 minutes at room temperature, washed twice with HBSS/1% bovine serum albumin (BSA) (Sigma‐Aldrich) and then blocked for 30 minutes with 5% BSA in HBSS at room temperature. Triton of 0.2% (Sigma‐Aldrich) was added during fixation to permeabilize the cells. Cells are incubated overnight (16 hours) at 4°C with primary antibodies: VWF‐Fitch (1 μg/mL, ab8822 Abcam, Cambridge, UK), VE‐cadherin (1 μg/mL, R&D Systems, MAB9381), Ang‐2 (1 μg/mL, AF623 R&D systems), P‐selectin (0.5 μg/mL, 556 087, BD, Franklin Lakes, New Jersey), Ki‐67 (0.5 μg/mL, 550 609, BD), and the appropriate control immunoglobulin G (IgG)1 or IgG2a isotype antibodies (X0931, Dako, Santa Clara, California; 559 319, BD) diluted in HBSS/1% BSA. After washing three times with HBSS/1% BSA, cells are incubated with Hoechst 33258 (H3569, Life Technologies, Thermo Fisher Scientific) and the appropriate secondary antibody (1:500) goat‐α‐mouse labeled with Alexa 488/568 (Molecular Probes, Eugene, OR, IgG1: A11001, A11004; IgG2a: A21134, A21131) for 2 hours at 4°C. Cells were again washed for three times with HBSS/1% BSA and covered with 1, 4‐diazobicyclo‐2,2,2‐octane (DABCC) glycerol. Cells were imaged using a Leica SP8 white light laser confocal immunofluorescence microscopy.

Sequential 12‐bit confocal images (*xyz* dimensions, 0.142 × 0.142 × 0.3, 0.142 × 0.142 × 1, or 0.116 × 0.116 × 1 μm) were recorded using Leica Application Suite X (LASX) Image software and analyzed with ImageJ. VWF was quantified as percentage of positive‐stained cells, defined as minimal of one clear group of pixels of VWF staining within cell, of the total cells per field of view. From each independent experiment (n = 4), 200 cells were analyzed.

### 2.6 Western blotting

After the hMVECs and hiPSC‐ECs reached a confluent state, they were lysed in lysis buffer (50 mM Tris‐HCl, 150 mM NaCl, 1% sodium deoxycholate (SDS), 0.5% Triton X‐100) supplemented with protease inhibitor (1:100). Sonoporation was used to achieve complete cell disrupture. The protein concentration was determined with a BCA protein kit (Thermo Fisher Scientific). Loading samples consisting of Red Pack loading buffer (New England Biolabs, Ipswich, Massachusetts), SDS‐polyacrylamide gel electrophoresis (PAGE), lysis buffer supplemented with protease inhibitor and 6.5 μg protein sample were incubated at 95° for 10 minutes. Proteins, transferred on a nitrocellulose membrane (Bio‐Rad, Hercules, California) were detected with antibodies against VWF (A0082 Dako), MYC (5605S Cell Signaling, Leiden, the Netherlands), MYCN (84 406, Cell Signaling), monocarboxylate transporter member 1 (MCT1; 20139‐1‐AP ProteinTech, Manchester, UK), and glyceraldehyde‐3‐phosphate dehydrogenase (GAPDH; DIGH11, Cell Signaling). After incubation, the membrane was washed and incubated with a horseradish peroxidase (HRP)‐conjugated secondary antibody (p0047, Dako) at room temperature for 1 hour. Afterward, signal was generated after 5 minutes of incubation in enhanced chemiluminescence (ECL) (Perkin Elmer, Waltham, MA) whereupon signal was emitted in a ChemicDoc Imaging System (Bio‐Rad).

The Simple Western Wes assay of ProteinSimple (Bio‐Techne, San Jose, California) was used to detect MCT1 (1:50, ProteinTech20139‐1‐AP) and GAPDH (1:20, DIGH11, Cell Signaling) according to the manufacturer's protocol using 0.2 μg/μl for each sample.^36^

### 2.7 RNA isolation and qPCR

After the hMVECs and hiPSC‐ECs reached a confluent state (at day 4), they were washed with Dulbecco's phosphate‐buffered saline (DPBS) whereupon Trizol (Ambion, Thermo Fisher Scientific) was added. RNA isolation was achieved using an RNeasy mini kit (Qiagen, Hilden, Germany), and quantitative polymerase chain reaction (qPCR) was performed as previously described.^35^ Forward and reversed VWF, KLF2, and MCT1 primer sequences are depicted in Table 1. Ct values were normalized by the Ct of GAPDH.

**Table 1:** Primer sequences

von Willebrand factor (VWF) primer sequence	
hu VWF forward	CCGATGCAGCCTTTTCGGA
hu VWF reverse	TCCCCAAGATACACGGAGAGG
Krüppel‐like factor 2 (KLF2) primer sequence	
hu KLF2 forward	CTACACCAAGAGTTCGCATCTG
hu KLF2 reverse	CCGTGTGCTTTCGGTAGTG
Monocarboxylate transporter 1 (MCT1) primer sequence	
hu MCT1 forward	AGTAGTTATGGGAAGAGTCAGCA
hu MCT1 reverse	GTCGGGCTACCATGTCAACA

### 2.8 RNA sequencing

Samples from three independent experiments were used for RNA sequencing. For each sample, an indexed cDNA library was prepared from 1 μg total RNA using the KAPA‐stranded mRNA‐seq kit (Sopachem, Ochten, the Netherlands). Clusters were generated using the Cbot2 system (Illumina, San Diego, California), and amplified cDNA fragments were sequenced on a HiSeq 4000 system (Illumina) as follows: 51 cycles for read 1 and 8 cycles for index 1. The raw sequenced reads were mapped to the human reference genome build GRCh38 using spliced transcripts alignment to a reference (STAR).^37^ Mapped reads were quantified using RSEM^38^ for accurate quantitation resulting in, on average, 34 740 890 ± 8 771 147 counts per sample. After autoscaling, the resulting data were first summarized by principal component analysis (PCA) using the flashPCA (R package). Plotly was used to generate interactive graphs (2D plots). Heatmap analysis was performed using the heatmaply package. RNA‐sequencing data are available in ArrayExpress (https://www.ebi.ac.uk/arrayexpress/experiments/E‐MTAB‐8392/) under accession E‐MTAB‐8392.

### 2.9 Lentiviral transductions

The human KLF2 overexpression construct was kindly provided by Prof. A. Horrevoets, VUMC Amsterdam, the Netherlands. Lentiviral particles were produced as described by the Sigma Library protocol using HEK293T cells. For transduction of hMVECs or iPSC‐ECs with the lentiviral expression vectors for *KLF2* ^*OE*^, mock cells were cultured to a 60%‐80% confluent state in EC‐SFM medium and transduced with the respective lentivirus in combination with 8 μg/mL polybrene (Sigma‐Aldrich) and incubated for 1 hour (37°C and 5% CO_2_). After 1 hour, the transduction medium was replaced with fresh EC‐SFM and incubated at 37°C and 5% CO_2_, for another 12 hours. Hereafter, the EC‐SFM medium was refreshed and the cells were incubated at 37°C and 5% CO_2_ for another 12 hours before collecting them for experimental assays.

### 2.10 VWF transfection

For transfection of hiPSC‐ECs with VWF plasmid (pcDNA 3.1[+] zeo human VWF‐MYC, provided by A. de Jong, LUMC, Leiden, the Netherlands^39^), cells were cultured to a 60%‐80% confluent state in EC‐SFM medium and transfected with transfection medium (Cell Applications Inc., San Diego, California, transfection kit) containing transfection medium, cytofect‐2 (CF2; 1:400), peptide enhancer (PE; 1:400), and VWF plasmid (300 ng/mL) for 1 hour at 37°C and 5% CO_2_. Hereafter, transfection medium was replaced for fresh EC‐SFM, and the cells were incubated for 24 hours (37°C, 5% CO_2_). Subsequently, the EC‐SFM medium was refreshed and after another 24 hours of incubation (37°C, 5% CO_2_), the cells were fixed.

### 2.11 Intracellular pH measurements

The cell‐permeant pH indicator, carboxy SNARF‐1 acetoxymethyl ester, acetate (Molecular Probe), was used to determine intracellular pH (pHi). As described previously,^40^ carboxy‐SNARF‐1 exhibits a significant pH‐dependent emission shift from yellow‐orange to deep‐red fluorescence under acidic and basic conditions, respectively, enabling the measurement of pH differences between pH 5 and 9. Cells were plated overnight on a black 96‐well plate, then loaded with SNARF‐1 for 30 minutes at 37°C and 5% CO_2_. For each cell type, a standard curve of pH values with nigericin (10 mM) was taken along. The pHi was determined by the ratio of the fluorescence intensities from the dye at two emission wavelengths—580 and 640 nm—measured by Molecular Devices Spectramax i3x. Cells were treated with 10 mM acetic acid (Merck, Burlington, Vermont) dissolved in culture medium (pH = 7.1) for 24 hours.

For alkalization of the intracellular compartments, cells were treated with 10 μM monensin (R&D systems) in EC‐SFM or EC‐SFM + HCL (pH = 7.0) and 10 μM nigericin (Sigma‐Aldrich) for 1 hour before fixation.

### 2.12 Proliferation measurement

To determine proliferation activity, ECs were seeded at a density of 20 000 cells in a 24‐well plate and left to adhere for 16 hours, followed by 24‐hour treatment with 10 mM acetic acid or EC‐SFM. Subsequently, cells were treated with 3‐(4,5‐dimethylthiazol‐2‐yl)‐2,5‐diphenyltetrazolium bromide (MTT, 5 mg/mL, Sigma Aldrich) 1:10 in culture medium and incubated for 2 hours at 37°C. Afterward, MTT was removed and isopropanol/0.04 M HCL was added and measured at 570 nm by Molecular Devices Spectramax i3x. Cellular protein content was determined with a BCA‐protein kit from Pierce (Thermo Fischer Scientific) and the data shown as MTT normalized to mg protein.

### 2.13 Analysis of metabolites

Quantitative analysis of intracellular and extracellular metabolites was performed using nuclear magnetic resonance (NMR) spectroscopy as described in detail elsewhere.^41^, ^42^ Briefly, hMVECs and hiPSC‐ECs in triplicate were washed with warm phosphate‐buffered saline (37°C) to remove the culture medium and quickly quenched with liquid nitrogen to arrest metabolism. The cells well subsequently scraped of the plates and extracted using a cold (−80°C) solution of methanol/chloroform/water, 8.1:0.9:1 (vol/vol/vol). After leaving the samples on dry ice for at least 30 minutes, the extracts were centrifuged for 20 minutes at 18 000*g* at −4°C.

Prior to washing cells with PBS, 0.2 mL of culture medium was collected from each sample and mixed with 0.4 mL of cold (−80°C) 100% liquid chromatography (LC)‐grade methanol to extract extracellular metabolites. All samples were subsequently placed at −80°C for at least 30 minutes and centrifuged for 20 minutes at 18 000g, at −4°C.

The supernatants from both cell extracts and culture medium extracts were collected and dried with nitrogen gas. NMR samples of extracts were prepared by dissolving the dried material with 0.22 mL of 0.15 M phosphate buffer (pH 7.4) in deuterated water containing 0.05 mM trimethylsilyl propionic‐*d*_4_‐sodium salt as internal standard for NMR referencing and quantification. An 1D ^1^H NMR spectrum was collected for each sample with a 14.1 T (600 MHz for ^1^H) Bruker Avance II NMR, using the 1D nuclear Overhauser effect spectroscopy (NOESY) experiment with presaturation as implemented in the spectrometer library (Topspin v3.0, pulse sequence: noesygppr1d; Bruker Biospin Ltd). All spectra were processed to correct the phase and baseline and imported in Chenomx NMR suite 8.4 (Chenomx NMR suite, v8.0, Edmonton, AB, Canada) for the quantification of metabolites. The protein pellet was dissolved in lysis buffer (150 mM NaCl, 1% SDS, 0.5% deoxycholate, and 0.5% Triton X‐100, pH 7.5) with sonification. The protein concentration was measured using PierceTM BCA protein assay kit (Thermo Fisher Scientific) according to its manual. All concentrations were normalized to the total protein mass of each sample.

For ^13^C fractional enrichment analysis, two triplicates of hMVEC and hiPSC‐ECs were cultured in parallel in either a glucose‐free medium, enriched by 5 mM U‐^13^C_6_‐d‐glucose for 24 hours or in medium with nonisotopically enriched d‐glucose at the same concentration (5 mM) and processed as described above. Fractional enrichment of ^13^C‐labeled metabolites was calculated using the differences between integrated areas of protons bonded to ^13^C from those bonded to ^12^C, as described elsewhere.^42^

Uptake or release of metabolites from or to the culture medium, respectively, was calculated as: ([*C*_spent_ − *C*_blank_] × *V*)/ protein, where *C*_spent_ is the concentration of metabolite in the culture medium (mmol/L), *C*_blank_ is the concentration in the cell‐free medium (mmol/L), and *V* (L) is the volume of culture medium in each petri dish. Negative values indicate uptake and positive values indicate release of metabolites.

### 2.14 Statistical analysis

Results are presented as mean ± SD or mean ± SEM, n defines the number of biological replicates. Differences between groups were assessed by nonpaired two‐tailed Student's *t* test, paired two‐tailed Student's *t* test or, when not normally distributed, by two‐tailed *F* test. Difference between >2 groups was assessed by analysis of variance (ANOVA) and Tukey's post hoc testing. *P* values <.05 were considered statistically significant.

## 3 RESULTS

### 3.1 hiPSC‐ECs express an immature endothelial genotype and phenotype

**Figure 1:**
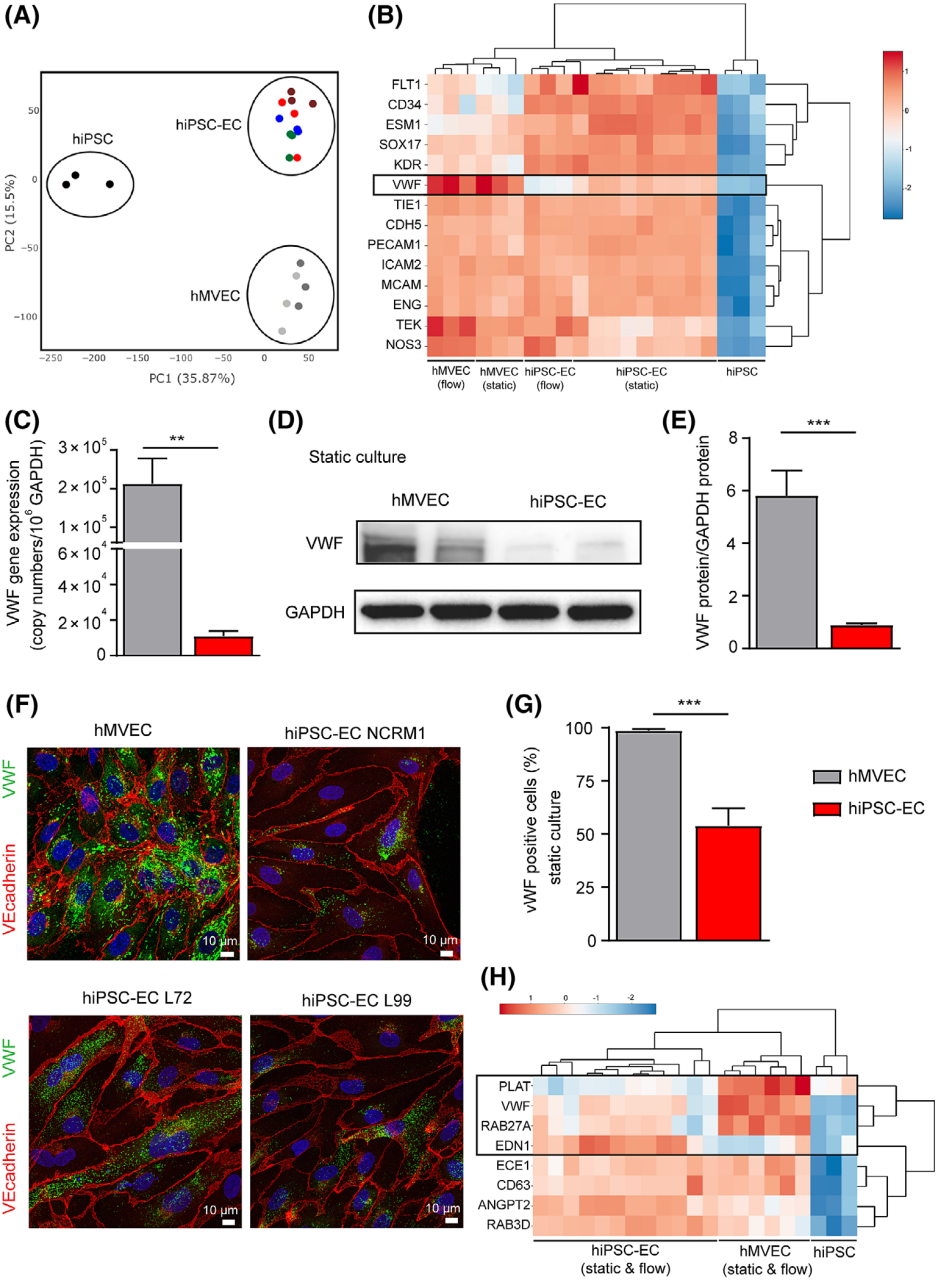
Assessment of hiPSC‐ECs maturity. A, Principle component analysis of all genes acquired from RNA‐sequencing results of hiPSC (black), mature ECs (hMVECs, grey) and hiPSC‐ECs in static conditions and after exposure to flow (dark grey: hMVEC and dark red: hiPSC‐EC NCRM1). Three hiPSC lines were used to create hiPSC‐ECs: NCRM1 (red), L72 (blue), L99 (green). B, Heatmap of 14 endothelial markers acquired from RNA‐sequencing results of hiPSC, mature ECs (hMVECs) and hiPSC‐ECs in static conditions and after exposure to flow. Scale bar represents *Z* scores: blue indicates lower gene expression and red, a higher gene expression. C, qPCR results of VWF expression of hiPSC‐ECs NCRM1 and hMVECs after static culture normalized by GAPDH. D, Western blot analyses of VWF protein expression of hMVECs and hiPSC‐ECs NCRM1 after static cell culture. GAPDH was included as a positive control. Blots are representative of three independent experiments. E, Quantification of western blot analyses of VWF protein of hMVECs and hiPSC‐ECs NCRM1 after static cell culture. F, Representative cross‐sectional confocal images stained for VWF (green) and VE‐cadherin (red) after static culture of hMVECs, hiPSC‐ECs NCRM1, hiPSC‐ECs L72, hiPSC‐ECs L99. G, VWF positive cells were quantified (n = 200/group). H, Heatmap of WPB storage components acquired from RNA‐sequencing results of hiPSC, mature ECs (hMVECs) and hiPSC‐ECs in static conditions and after exposure to flow. Scale bar represents *Z* scores: blue indicates lower gene expression and red, a higher gene expression. Values are given as mean ± SEM of three to four independent experiments. Nonpaired two‐tailed Student's *t* test was performed; *P < .05, **P < .001, ***P < .0001. GAPDH, glyceraldehyde‐3‐phosphate dehydrogenase; hiPSC‐ECs, human‐induced pluripotent stem cell‐derived endothelial cells; hMVECs, human microvascular ECs; VWF, von Willebrand factor; WPB, Weibel‐Palade body

First, we investigated the maturity of hiPSC‐ECs compared with control primary hMVECs and their undifferentiated precursor cells (hiPSCs) by assessing a whole genome RNA expression profile. PCA demonstrated a distinct clustering of the three cell types, indicating different transcriptome profiles (Figure 1A). Heatmap analysis of endothelial markers revealed increased expression of the early progenitor and stem cell markers *CD34* and *SOX17* in hiPSC‐ECs and a reduced VWF gene expression (Figure 1B), indicating an immature EC phenotype. qPCR analysis was performed to confirm *VWF* mRNA expression of the hiPSC‐ECs compared with hMVECs under static conditions (Figure 1C). As demonstrated before, *VWF* gene expression of hiPSC‐ECs is remarkably reduced compared with hMVECs, which is also reflected by lower VWF protein in hiPSC‐ECs (Figure 1D,E). We next compared cell morphology of hiPSC‐ECs derived from three different iPSC lines (NCRM1, L72, and L99) to control for cell line‐specific effects to hMVECs under static culture conditions. The hMVECs showed abundancy of elongated WPBs (Figure 1F), where the hiPSC‐ECs showed reduced and punctate VWF (Figure 1F). Only ~50% of the hiPSC‐ECs were positive for VWF presence (Figure 1G). In addition, the RNA‐sequencing results of WPB storage components further revealed downregulation of Ras‐related protein RAB‐27A (*RAB27A*) and tissue‐type plasminogen activator (*PLAT*) in hiPSC‐ECs and upregulation of *EDN1* and *ANGPT‐2* when compared with hMVECs (Figure 1H). To validate protocol‐independent immaturity of hiPSC‐EC WPB, we compared our results with the accessible RNA‐sequencing data of hiPSC‐ECs of Wimmer et al^1^ and Gu et al.^43^ For all genes (Figure S1A) and metabolic genes (Figure S1B), our results,^34^ cluster with the hiPSC‐EC data from Wimmer et al^1^ and Gu et al.^43^ Additionally, the primary ECs used cluster together and differ from the hiPSC‐EC. This analysis indicates that independent of the protocol used, differences in primary ECs and hiPSC‐EC genotype can be found for each hiPSC‐EC differentiation protocol.

### 3.2 Formation of WPB is not induced by shear stress, coculture, or KLF2^*OE*^/VWF^*OE*^

**Figure 2:**
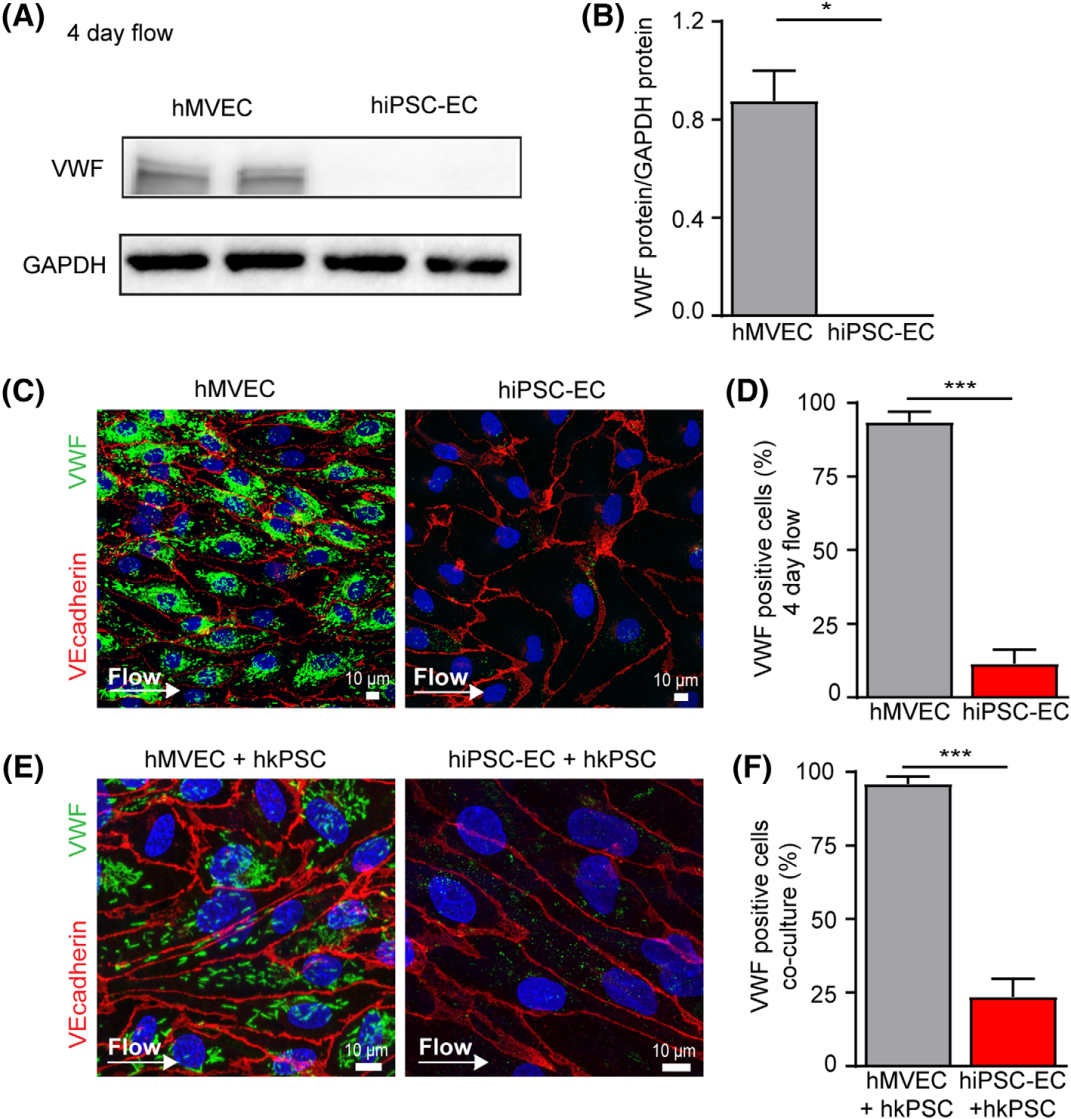
Shear stress and pericyte coculture do not induce VWF maturation in hiPSC‐ECs. A, Western blot analyses of VWF protein expression in hMVECs and hiPSC‐ECs NCRM1 after 4 days of laminar flow exposure. Blots are representative of three independent experiments. B, Quantification of western blot analyses of VWF protein of hMVECs and hiPSC‐ECs NCRM1 after 4 days of laminar flow exposure. C, Representative cross‐sectional confocal images stained for VWF (green) after 4 days of laminar flow of hMVECs and hiPSC‐ECs NCRM1. D, VWF positive cells were quantified (n = 200/group). E, Representative cross‐sectional confocal images stained for VWF (green) after 4 days of laminar flow and coculture of hMVECs and hiPSC‐ECs NCRM1 with hkPSCs. F, VWF‐positive cells were quantified (n = 200/group). Values are given as mean ± SEM of three to five independent experiments. Nonpaired two‐tailed Student's *t* test was performed; *P < .05, **P < .001, ***P < .0001. hiPSC‐ECs, human‐induced pluripotent stem cell‐derived endothelial cells; hMVECs, human microvascular ECs; VWF, von Willebrand factor

Prolonged laminar flow upregulates endothelial markers, like FLT1, TEK, and NOS3 in hMVECs (Figure 1B). However, VWF gene and protein expression in hiPSC‐ECs are strongly downregulated upon exposure to flow (Figures 2A,B and S2A). Compared with static conditions, flow exposure did not increase VWF and Ang‐2 staining in hiPSC‐ECs (Figures 2C and S2A,B) and only 12% of the hiPSC‐ECs were positive for the presence of WPBs, significantly lower compared with hMVECs (Figure 2D). Testing the role of hkPSCs in hiPSC‐ECs maturation also did not result in formation of WPBs or a notable increase of VWF, which remains as a punctate perinuclear staining in a fraction of cells (Figure 2E). Similarly, about 25% of the hiPSC‐ECs in coculture were positive for VWF presence (Figure 2F).

Shear stress, which chronically induces KLF2, directly can affect the production of VWF and, in turn, WPB. Overexpression of KLF2 mRNA (*KLF2* ^*OE*^) was confirmed by qPCR; however, this was without effect on VWF gene expression (Figure S2A). *KLF2* ^*OE*^ resulted in a subtle, but significant increase in VWF protein expression in hiPSC‐ECs (Figure S3B,C). However, confocal imaging of VWF staining revealed that lentiviral *KLF2* ^*OE*^ in hiPSC‐ECs did not affect WPB formation in hiPSC‐ECs as both mock and hiPSC‐ECs *KLF2* ^*OE*^ cells show a similar level of punctate VWF staining (Figure S3D,E). Finally, VWF transfection was performed to investigate whether WPB immaturity in hiPSC‐ECs could be a result of a limited VWF protein presence in these cells. Confocal data showed an increase in VWF staining intensity, however, did not improve WPB formation in VWF‐transfected hiPSC‐ECs (Figure S3F).

### 3.3 VWF tubulation and WPB elongation are dependent on low pHi

**Figure 3:**
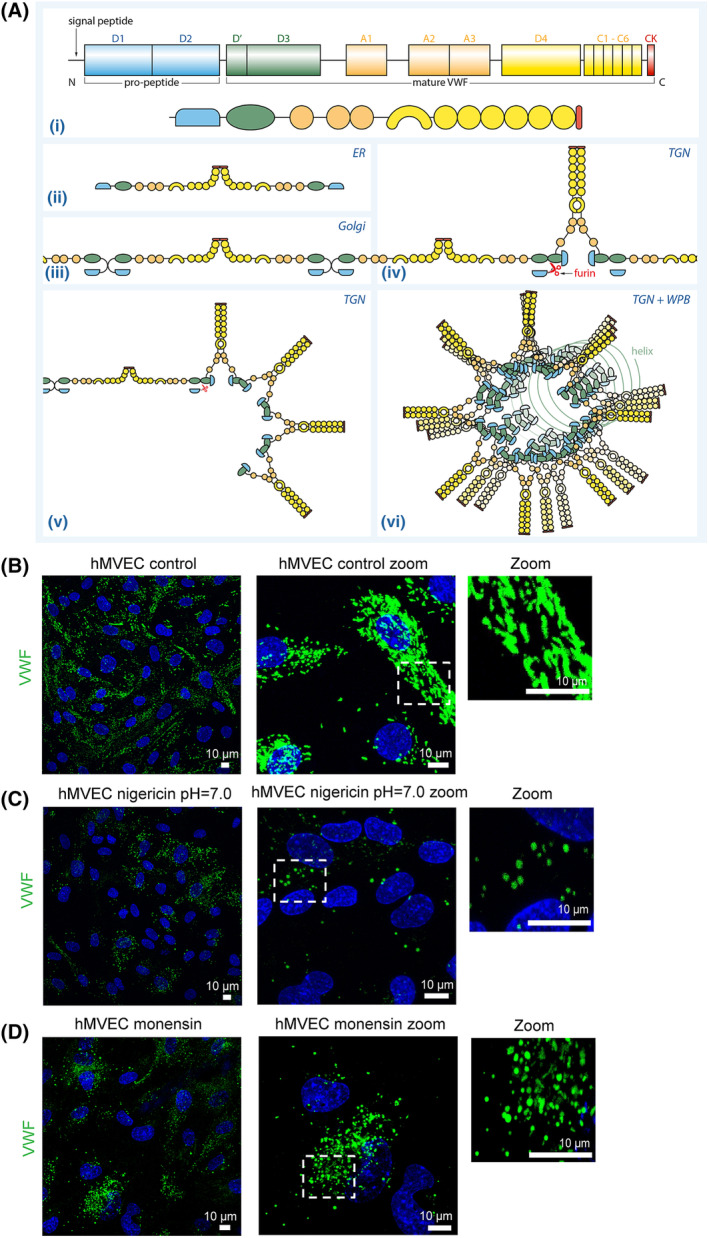
The shape and size of WPBs is pH dependent. A, Block diagram showing the domains of propeptide (D1 and D2), and the regions of the mature protein (D′‐CK) involved in dimerization. (i) N, N‐terminus; C, C‐terminus. (ii) C‐terminal dimerization in ER. (iii) N‐terminal intrachain disulfide bonds form in the Golgi apparatus. (iv,v) N‐terminal interchain disulfide bonds replace intrachain disulfide bonds and furin cleavage liberates domains D1 and D2 that remain associated with the mature VWF peptide at the acidic pH of the Golgi and WPB. Helical arrangement of the multimeric VWF tubules found in WPB. The N‐terminal domains (D1‐D2 and D'‐D3) form a helix whereas A2‐CK fold into a dimeric bouquets, both processes are pH regulated. (vi) The sequences neighboring A1 are likely to function as a flexible hinge allowing the C‐terminal part (A2‐CK) to pack between adjacent VWF tubules. Colors of the domains as in (i). Representative cross‐sectional confocal images stained for VWF (green) (n = 3). B, hMVECs cultured in normal medium (EC‐SFM). C, hMVECs cultured in pH = 7.0 medium (EC‐SFM + HCl) containing 10 μM nigericin 1 hour before fixation. D, hMVECs cultured in medium (EC‐SFM) containing 10 μM monensin 1 hour before fixation. CK, cysteine knot; EC‐SFM, endothelial cell serum‐free medium; ER, endoplasmic reticulum; hMVECs, human microvascular ECs; VWF, von Willebrand factor; WPBs, Weibel‐Palade bodies

The formation of WPBs is dependent on VWF tubulation by intrachain disulfide bond formation and association of the propeptide, after furin cleavage,^44^ with the mature VWF peptide at the acidic pH of the Golgi, TGN, and WPB (Figure 3A).^14^, ^16^, ^20^, ^45^, ^46^, ^47^, ^48^ Both dimeric bouquets formation and helical arrangement of VWF are pH regulated (Figure 3A).^23^, ^49^ Given the requirement of an acidic intracellular environment for this process, we questioned whether lack of elongated WPBs in hiPSC‐ECs could be due to an altered pHi regulation. The elongated “cigar” shape of WPBs in hMVECs (Figure 3B) indeed disappeared after the cells were exposed to nigericin, an antibiotic acting as an antiporter of H+ and K+ and thereby disturbing pHi maintenance, in medium of pH = 7.0 (Figure 3C).^14^ Similar results were obtained after 1‐hour appliance of culture medium‐containing monensin (10 μM), an ionophore that increases pHi by forming lipophilic complexes with monovalent cations to induce Na + influx and H^+^/K^+^ efflux, which results in alkalization of acidic intracellular compartments such as the Golgi apparatus^50^ (Figure 3D). These data suggest that an acidic pHi is indeed necessary for VWF tubulation, which in turn drives WPB elongation.

### 3.4 High pHi of hiPSC‐ECs impairs WPB formation

**Figure 4:**
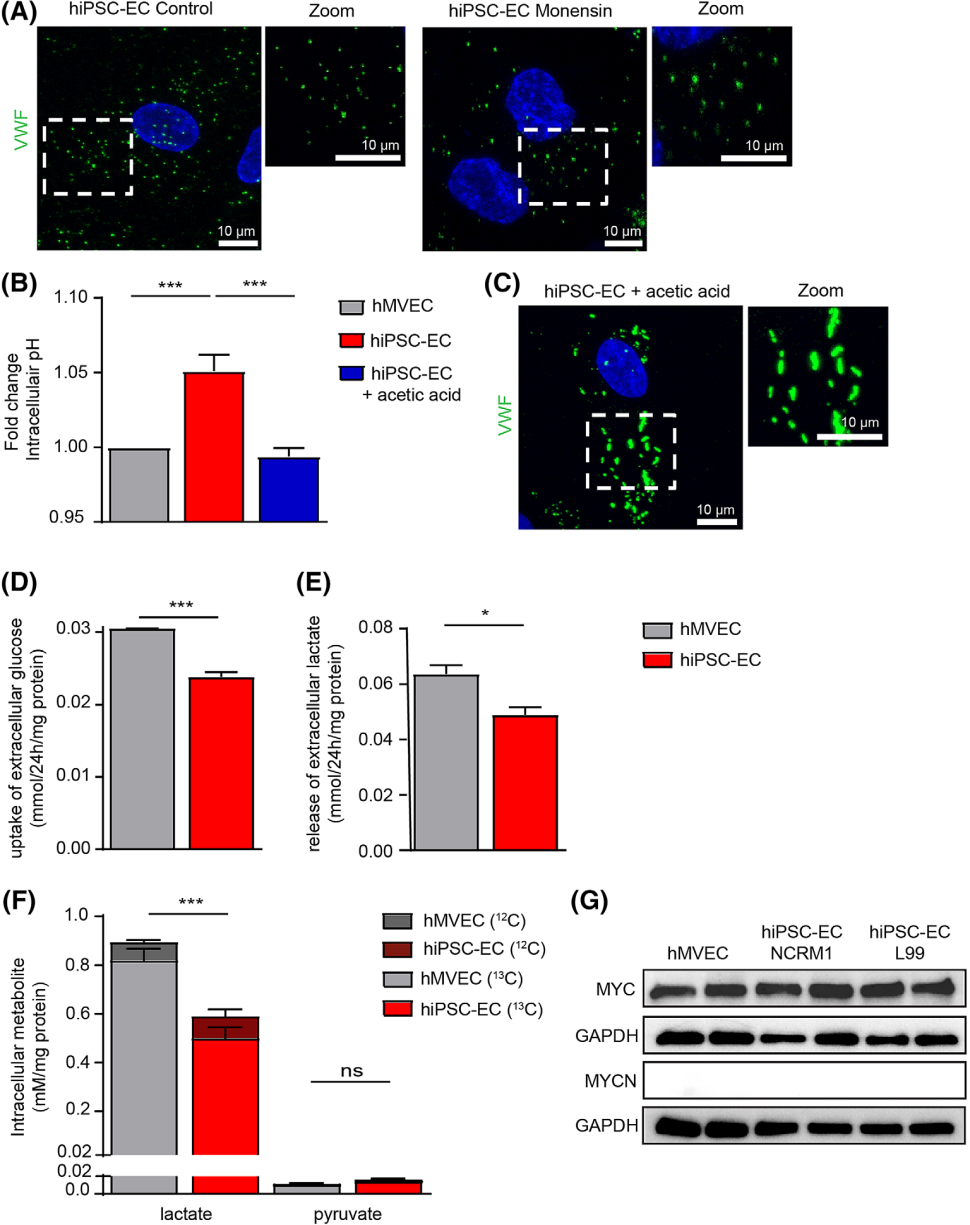
High intracellular pH of hiPSC‐EC, caused by low glycolysis, limits WPB formation. A, Representative cross‐sectional confocal images stained for VWF (green) of hiPSC‐ECs NCRM1 under normal conditions and after 1‐hour incubation with 10 μM monensin (n = 3). B, pHi of hiPSC‐ECs NCRM1 and hMVECs under normal conditions and after 24 hour incubation with acetic acid (10 mM) (n = 6). C, Representative cross‐sectional confocal images stained for VWF (green) hiPSC‐ECs NCRM1 cultured in acetic acid (10 mM) containing medium. D, Glucose uptake rate and, E, lactate release rate after 24 hours culture. F, Total intracellular concentrations of lactate and pyruvate and their ^13^C‐enriched fractions after incubation of cells in ^13^C_6_‐d‐glucose‐containing culture medium for 24 hours. G, Western blot analyses of MYC and MYCN protein expression in hMVECs, hiPSC‐ECs NCRM1 and hiPSC‐ECs L99. GAPDH was included as a positive control. Values are given as mean ± SEM of 3‐6 independent experiments. One‐way ANOVA and nonpaired two‐tailed Student's *t* test were performed; *P < .05, **P < .001, ***P < .0001. ANOVA, analysis of variance; GAPDH, glyceraldehyde‐3‐phosphate dehydrogenase; hiPSC‐ECs, human‐induced pluripotent stem cell‐derived endothelial cells; hMVECs, human microvascular ECs; ns, nonsignificant; pHi, intracellular pH; VWF, von Willebrand factor; WPBs, Weibel‐Palade bodies

The remarkable resemblance of hMVECs WPB phenotype, after increasing the pHi, to the hiPSC‐ECs WPBs made us question whether lowering the pHi of the hiPSC‐ECs could induce WPB formation in these cells. Addition of monensin, which directly increases pHi, to hiPSC‐ECs cell culture medium did not result in a different appearance of the WPBs, indicating that the pHi of hiPSC‐ECs was already too high to efficiently form WPBs (Figure 4A). By measuring the pHi with the cell permeable pH indicator SNARF‐1, we confirmed that pHi in hiPSC‐ECs is higher compared with hMVECs (Figure 4B). To stimulate VWF tubulation in hiPSC‐ECs, we lowered pHi using 10 mM acetic acid, which was confirmed by pHi measurement (Figure 4B). After lowering the pHi with acetic acid for 24 hours, mature elongated “cigar”‐shaped WPBs can be observed in the hiPSC‐ECs (Figure 4C) and were positive for VWF, Ang‐2, and P‐selectin (Figure S4A,B). This effect on the formation of WPB was independent of the amount of VWF protein, which did not significantly change upon addition of the acid (Figure 4C,D). Acetic acid addition resulted in an acidification of the culture medium to pH = 7.1, causing limited stress to the cells, such as minimal increase in focal adhesion junction, without a change in Ki‐67 expression (Figure 4E‐H). However, hiPSC‐EC treated with acetic acid showed a significant increase in proliferation, normalizing the proliferation to the level of primary ECs. In hMVEC, addition of acetic acid did not change the proliferation (Figure 4F).

### 3.5 Low rate of glycolysis could be causal of a high pHi

To understand the mechanism causing an increased pHi and lack of WPB maturation, we performed nuclear magnetic resonance (NMR) analysis to determine the cellular release and uptake of metabolites. hiPSC‐ECs were found to have reduced glucose uptake and lactate release, indicating lower glycolysis (Figure 4D,E). This was also reflected in the total intracellular lactate concentration which was significantly lower in hiPSC‐ECs as compared with hMVECs (Figure 4F). Carrying out ^13^C‐glucose tracer experiments, we determined the intracellular fate of glucose‐derived metabolites.^41^, ^42^ Of the total intracellular lactate, the ^13^C‐lactate fraction was lower in hiPSC‐ECs (81.6% ± 1.3%) compared with hMVECs (90.8% ± 0.7%), demonstrating a reduced glycolytic flux toward lactate (Figure 4F). Although oxidative phosphorylation of glucose is at least 20‐fold more efficient than glycolysis in producing adenosine triphosphate (ATP) per mole of glucose, less than 1% of pyruvate is oxidized through the tricarboxylic acid cycle (TCA) cycle by ECs in vitro, while 99% is converted to lactate through glycolysis.^51^, ^52^, ^53^ Both hiPSC‐ECs and hMVECs were found to convert most glucose into lactate; however hiPSC‐ECs do this at a significant lower rate (Figure 4D‐F). Glycolysis uncoupled from glucose oxidation, resulting in production of 2H^+^ from glycolytically derived ATP hydrolysis,^54^, ^55^ is a major cause of intracellular acidosis.^56^ Taken together, this could partially explain the observed higher pHi in hiPSC‐ECs (Figure 4B).

### 3.6 Dysregulation of glycolysis not caused by disturbed MYC and MYCN expression

The regulation of glycolysis in (stem) cell development is regulated by the balance between MYC and MYCN activity. Elevated glycolysis as observed in hiPSCs requires elevated MYC and MYCN activity.^57^ Metabolic switching during mesodermal differentiation coincides with a reduction in MYC and MYCN.^57^ Since ECs also mainly rely on glycolysis, MYC expression has to be switched on again, which was indeed observed in hiPSC‐ECs at a similar level as in hMVECs (Figure 4G), eliminating this balance in expression as a cause for the reduced glycolysis.

### 3.7 pHi‐sensitive lactate/H+ transporter MCT1 downregulated in hiPSC‐EC

**Figure 5:**
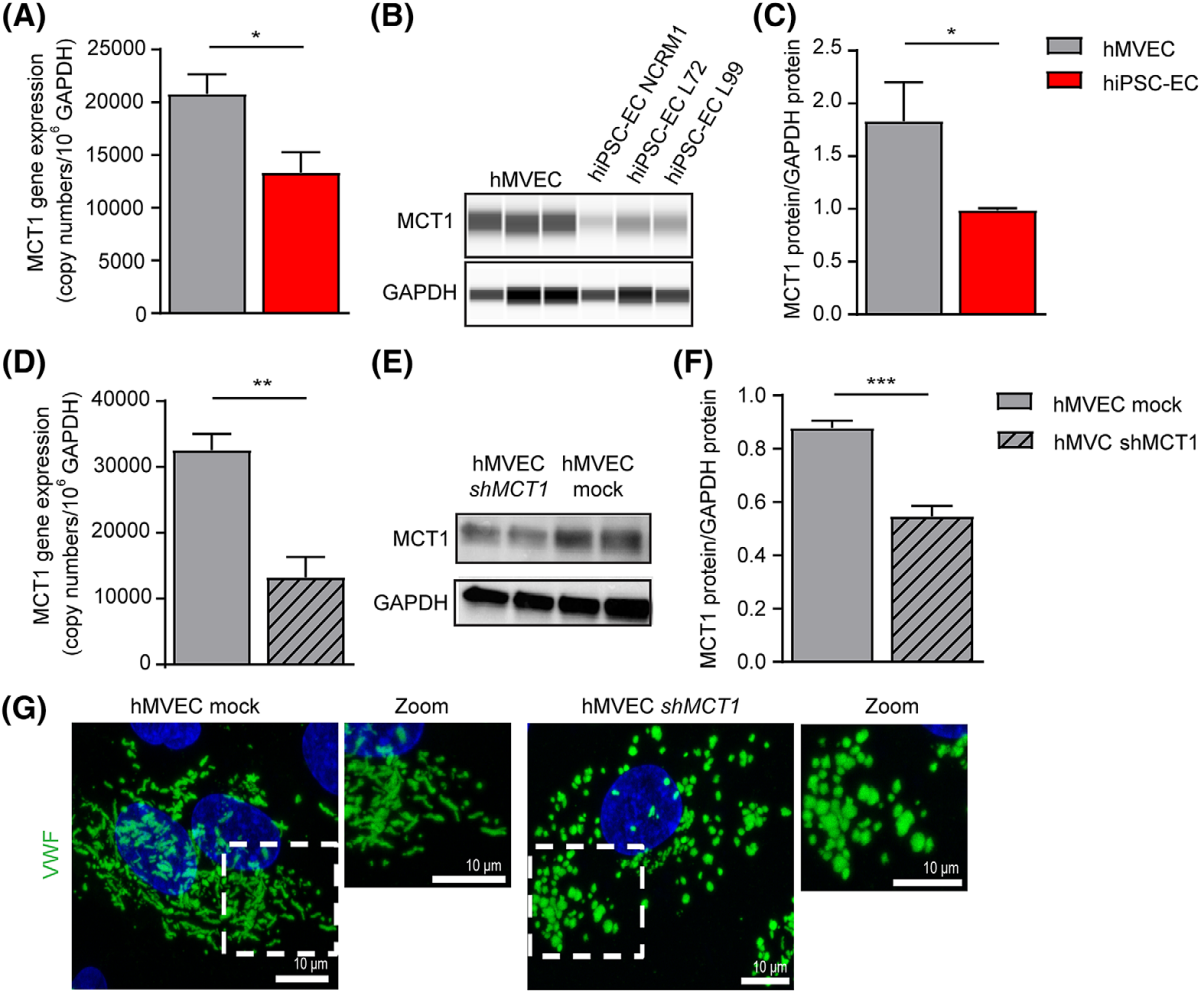
MCT1, a lactate/proton transporter, is involved in WBP maturation. A, qPCR results of MCT1 expression of hiPSC‐ECs NCRM1 and hMVECs. B, Simple Western Wes analysis of MCT1 protein expression in hMVECs and hiPSC‐ECs NCRM1, L72, and L99. GAPDH was included as a positive control. Blots are representative of three independent experiments. C, Quantification of Wes analysis of MCT1 protein expression in hMVECs and hiPSC‐ECs NCRM1, L72, and L99. GAPDH was included as a positive control. D, qPCR results of MCT1 expression of hMVECs mock and lentiviral MCT1 transduced cells. E, Western blot analyses of MCT1 protein expression in hMVECs mock and after MCT1 transduction. GAPDH was included as a positive control. Blots are representative of three independent experiments. F, Quantification of western blot analyses of MCT1 protein expression in hMVECs mock and MCT2 transduced cells. GAPDH was included as a positive control. G, Representative cross‐sectional confocal images stained for VWF (green) of hMVECs mock and lentiviral MCT1 transduced cells. Values are given as mean ± SEM of three to four independent experiments. Nonpaired two‐tailed Student's *t* test were performed; *P < .05, **P < .001, ***P < .0001. GAPDH, glyceraldehyde‐3‐phosphate dehydrogenase; hiPSC‐ECs, human‐induced pluripotent stem cell‐derived endothelial cells; hMVECs, human microvascular ECs; MCT1, monocarboxylate transporter member 1; VWF, von Willebrand factor; WPB, Weibel‐Palade body

Intracellular lactate and H+ concentration is not only determined by glycolytic flux but also by the monocarboxylate transporter MCT1, which serves as the main gate for lactate/H^+^ entry in ECs^58^, ^59^ In addition to the reduced glycolysis as a cause for low intracellular lactate/H+, we also observed a ~50% downregulation of MCT1 gene expression in hiPSC‐ECs (Figure 5A). These results were confirmed at the protein level (Figure 5B,C). MCT1 is known to be directly regulated by pHi; however, the alkalic pHi of iPSC‐EC does not cause sufficient induction of MCT1 to regulate H+ influx.^60^ To verify the involvement of the MCT1 downregulation in disturbed tubulation of VWF, we downregulated MCT1 in hMVECs by transduction with a short‐hairpin MCT1 construct (shMCT1). This resulted in a ~60% decrease in MCT1 gene expression (Figure 5D) and protein (Figure 5E,F), comparable with the downregulation in hiPSC‐ECs. Confocal microscopy revealed similar tubulation problems of the WPBs as increased round‐ and irregular‐shaped WPBs can be observed in *shMCT1*‐hMVECs compared with hMVECs mock (Figure 5G).

**Figure 6:**
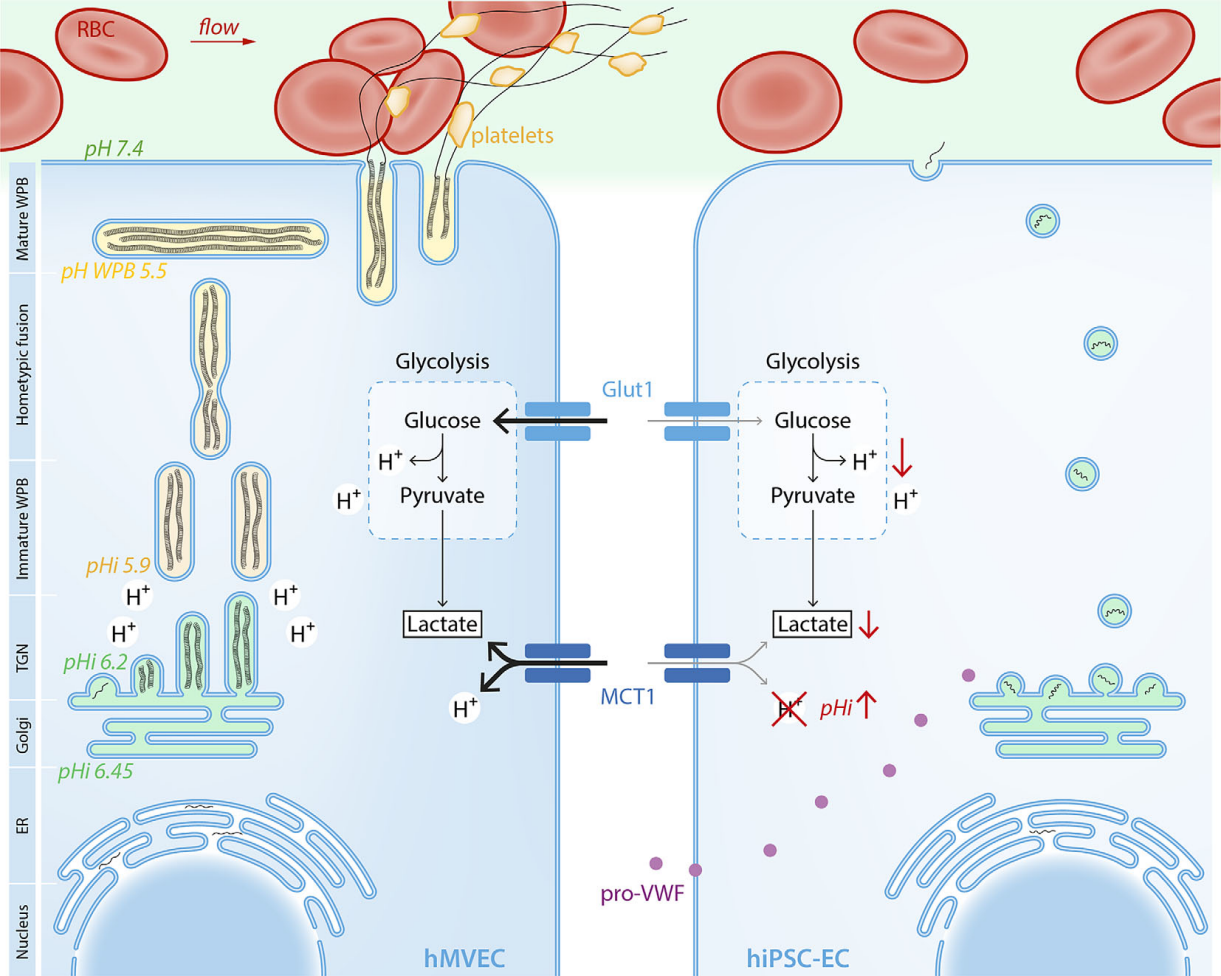
Schematic overview of WPB production and the metabolic regulation of intracellular pH (pHi) of hMVECs and hiPSC‐ECs. Formation of mature WBP in hiPSC‐ECs is limited by the increased intracellular pH around the Golgi apparatus and TGN. This increase in pHi is caused by reduced intracellular lactate accompanied by reduced H^+^, caused by reduced glycolysis and reduced uptake of lactate and H^+^ via MCT1. This increased pHi limits intrachain disulfide bonds formation and association of the propeptide, after furin cleavage, with the mature VWF peptide at the TGN and WPB. Both dimeric bouquets formation and helical arrangement of VWF are pH regulated. Eventually this reduces the amount of WPB and limits the functionality of the endothelial cell. hiPSC‐ECs, human‐induced pluripotent stem cell‐derived endothelial cells; hMVECs, human microvascular ECs; MCT1, monocarboxylate transporter member 1; TGN, *trans*‐Golgi network; VWF, von Willebrand factor; WPB, Weibel‐Palade body

To conclude, the formation of mature WPB in hiPSC‐ECs is limited by increased pHi caused by metabolic dysfunction, such as reduced glycolysis and limited expression of the lactate/H+ transporter MCT1 (Figure 6).

## 4 DISCUSSION

Here we show that formation of mature WPB in hiPSC‐ECs is limited by increased pHi around the Golgi apparatus and TGN. This increase in pHi is caused by reduced intracellular lactate accompanied by reduced H^+^, as a result of reduced glycolytic flux and reduced cellular uptake of lactate and H^+^ via the MCT1 (Figure 6). The resulting increased pHi is known to limit intrachain disulfide bonds formation and association of the propeptide, after furin cleavage,^44^ with the mature VWF peptide at the TGN and WPB.^14^, ^16^, ^20^, ^45^, ^46^, ^47^, ^48^ Both dimeric bouquets formation and helical arrangement of VWF are pH regulated.^23^, ^49^ The hiPSC‐ECs WPB maturation can be rescued by acetic acid supplementation, which directly lowered pHi.

Given the critical role of ECs in angiocrine signaling,^61^ and oxygen and energy transport, the generation of functional hiPSC‐ECs is a crucial step in the field of stem cell‐based disease modeling and regenerative medicine. The current study shows that hiPSC‐ECs express an immature endothelial genotype and phenotype, which do not improve after exposure to shear stress or KLF2 overexpression. Our data point to a critical role for persisting altered cell metabolism and downstream effects on pHi as the main culprit for WPB maturation failure. Indeed, pluripotent stem cell fate decisions are made through regulation of metabolic flux, where a high rate of anaerobic glycolysis confers stemness.^57^ To allow for specification into mesodermal lineages, such as ECs, glycolysis is switched to oxidative phosphorylation through reduction of MYC and MYCN activity. Interestingly, by contrast, mature ECs are characterized by a high glycolytic flux.^52^ Our data suggest that this latter adaptation does not fully develop in the EC differentiation protocol, despite increased MYC expression necessary for enhanced glycolysis after MYC and MYCN downregulation during mesoderm differentiation.

Another factor that may have contributed to WPB maturation failure is the observed downregulation of the MCT1 in hiPSC‐ECs. MCTs 1‐4 are known to facilitate transmembrane proton cotransport of monocarboxylates such as lactate, pyruvate, acetoacetate, and β‐hydroxybutyrate across the cell membrane.^62^ Because MCT1 is responsible for the import of lactate and H^+^,^47^ downregulation of MCT1 in hiPSC‐ECs could explain the lower uptake and intracellular concentration of both protons (pHi) and lactate. Lactate is involved in multiple regulatory functions of the cell, including transcriptional stimulation of MCT1, thus coupling reduced glycolytic flux to MCT1 downregulation.^62^, ^63^

Generally, the amount of structural and enzymatic components will determine the size or number of organelles.^64^ For WPBs, it is well established that the cargo protein VWF is the driver for WPB formation.^22^ Gradual reduction in VWF cellular content by siRNA correlated with a gradual decrease in the number of WPBs and, more importantly, also shortening of WPB length.^65^, ^66^ hiPSC‐ECs were found to have very low gene expression of VWF and limited amount of VWF protein. Exposing hiPSC‐ECs to shear stress did not result in VWF upregulation, probably caused by the reduced glycocalyx surface coat and consequently impaired shear sensing of hiPSC‐ECs.^34^ Recent work on hiPSC‐ECs in 3D models showed improved maturation of hiPSC‐ECs and differentiation into various types of ECs (venular, capillary, arteriolar, arterial) upon transplantation in mice; however, VWF expression of these ECs in both 2D and 3D models remained surprisingly low.^1^

Although direct upregulation of VWF by *VWF* ^*OE*^ or *KLF* ^*OE*^ resulted in a slight increase in VWF protein in hiPSC‐ECs, WPBs were still limited and round. This underlines the involvement of a posttranscriptional modification problem caused by the metabolic immaturity of hiPSC‐ECs. Although we demonstrated that decreasing the pHi in hiPSC‐ECs induced WPB maturation without an increase in VWF gene expression, the low amount of VWF protein potentially plays a role in the WPB formation. Assessment of combinatory effects of both VWF^*OE*^ to induce VWF production and addition of acidic acid to restore WBP formation would be of further interest to optimize the function of WPBs in hiPSC‐ECs.

Apart from VWF, several other proteins that regulate endothelial function can be recruited to the WPBs.^11^, ^18^, ^20^, ^25^, ^26^, ^27^ The composition of the WPB is essential to EC organotypic function; for example, in lung ECs, WPB contain clotting factor VIII. The exact composition also determines processes such as vessel stability, where Ang‐2 derived from WPBs may dysregulate pericyte stabilization of ECs and induce angiogenesis.^67^, ^68^

## 5 CONCLUSIONS

Although differentiation of hiPSCs into endothelial‐like cells (hiPSC‐ECs) has been demonstrated before, hiPSC‐ECs fail to express mature WPBs. Maturation of WPB is a key requirement to generate functional EC from iPSC, as WPBs are essential for angiogenesis, primary and secondary hemostases. Our data show that such WPB maturation failure can be rescued in cell culture by lowering the increased pHi by adding acetic acid. Along the same lines, one can speculate about similar strategies that reduce pH or increase glycolytic flux in ECs in organoid cultures to achieve tissue maturation.^69^

## CONFLICT OF INTEREST

J.C.J.E. declared research funding from CSL Berhing. The other authors declared no potential conflicts of interest.

## AUTHOR CONTRIBUTIONS

G.L.T., R.d.K., B.M.v.d.B.: concept and design, collection/assembly of data, data analysis and interpretation, manuscript writing; G.W., S.D., R.G.J.R., S.K.: collection/assembly of data, data analysis and interpretation; W.M.P.J.S.: collection of data; C.v.d.B., M.G., J.C.J.E.: read the manuscript, provided helpful comments; P.C.: financial support, read the manuscript, provided helpful comments; T.J.R.: concept and design, manuscript writing, financial support.

## Supporting information


**Appendix** **S1**: Supplemental data
**Figure S1.** Comparison of hiPSC‐EC differentiation protocols by RNA‐sequencing
**Figure S2.** Lack of Weibel‐Palade bodies in hiPSC‐ECs
**Figure S3.** hiPSC‐ECs WPB phenotype of KLF2 transduced cells
**Figure S4.** Effects of acetic acid addition in hiPSC‐ECs

## Data Availability

The RNA‐sequencing data that support the findings of this study are openly available at ArrayExpress (https://www.ebi.ac.uk/arrayexpress/experiments/E-MTAB-8392/) under accession number E‐MTAB‐8392.

## References

[1] Wimmer RA , Leopoldi A , Aichinger M , et al. Human blood vessel organoids as a model of diabetic vasculopathy. Nature. 2019;565(7740):505‐510. 30651639 10.1038/s41586-018-0858-8 PMC7116578

[2] Ong SB , Lee WH , Shao NY , et al. Calpain inhibition restores autophagy and prevents mitochondrial fragmentation in a human iPSC model of diabetic endotheliopathy. Stem Cell Rep. 2019;12(3):597‐610. 10.1016/j.stemcr.2019.01.017 PMC6411483 30799273

[3] Orlova VV , van den Hil FE , Petrus‐Reurer S , Drabsch Y , Ten Dijke P , Mummery CL . Generation, expansion and functional analysis of endothelial cells and pericytes derived from human pluripotent stem cells. Nat Protoc. 2014;9(6):1514‐1531. 24874816 10.1038/nprot.2014.102

[4] Rufaihah AJ , Huang NF , Jame S , et al. Endothelial cells derived from human iPSCS increase capillary density and improve perfusion in a mouse model of peripheral arterial disease. Arterioscler Thromb Vasc Biol. 2011;31(11):e72‐e79. 21836062 10.1161/ATVBAHA.111.230938 PMC3210551

[5] Rufaihah AJ , Huang NF , Kim J , et al. Human induced pluripotent stem cell‐derived endothelial cells exhibit functional heterogeneity. Am J Trans Res. 2013;5(1):21‐35. PMC3560482 23390563

[6] Li Z , Hu S , Ghosh Z , Han Z , Wu JC . Functional characterization and expression profiling of human induced pluripotent stem cell‐ and embryonic stem cell‐derived endothelial cells. Stem Cells Dev. 2011;20(10):1701‐1710. 21235328 10.1089/scd.2010.0426 PMC3182033

[7] Park SW , Jun Koh Y , Jeon J , et al. Efficient differentiation of human pluripotent stem cells into functional CD34+ progenitor cells by combined modulation of the MEK/ERK and BMP4 signaling pathways. Blood. 2010;116(25):5762‐5772. 20884805 10.1182/blood-2010-04-280719

[8] Halaidych OV , Freund C , van den Hil F , et al. Inflammatory responses and barrier function of endothelial cells derived from human induced pluripotent stem cells. Stem Cell Rep. 2018;10(5):1642‐1656. 10.1016/j.stemcr.2018.03.012 PMC5995303 29657098

[9] Adams WJ , Zhang Y , Cloutier J , et al. Functional vascular endothelium derived from human induced pluripotent stem cells. Stem Cell Rep. 2013;1(2):105‐113. 10.1016/j.stemcr.2013.06.007 PMC3757754 24052946

[10] Deanfield John E , Halcox Julian P , Rabelink TJ . Endothelial function and dysfunction. Circulation. 2007;115(10):1285‐1295. 17353456 10.1161/CIRCULATIONAHA.106.652859

[11] Nightingale T , Cutler D . The secretion of von Willebrand factor from endothelial cells; an increasingly complicated story. J Thromb Haemost. 2013;11(Suppl 1):192‐201. 10.1111/jth.12225 PMC4255685 23809123

[12] Valentijn KM , Sadler JE , Valentijn JA , Voorberg J , Eikenboom J . Functional architecture of Weibel‐Palade bodies. Blood. 2011;117(19):5033‐5043. 21266719 10.1182/blood-2010-09-267492 PMC3109530

[13] Leebeek FW , Eikenboom JC . Von Willebrand's disease. N Engl J Med. 2016;375(21):2067‐2080. 27959741 10.1056/NEJMra1601561

[14] Michaux G , Abbitt KB , Collinson LM , Haberichter SL , Norman KE , Cutler DF . The physiological function of von Willebrand's factor depends on its tubular storage in endothelial Weibel‐Palade bodies. Dev Cell. 2006;10(2):223‐232. 16459301 10.1016/j.devcel.2005.12.012

[15] Lenting PJ , Christophe OD , Denis CV . von Willebrand factor biosynthesis, secretion, and clearance: connecting the far ends. Blood. 2015;125(13):2019‐2028. 25712991 10.1182/blood-2014-06-528406

[16] Springer TA . von Willebrand factor, Jedi knight of the bloodstream. Blood. 2014;124(9):1412‐1425. 24928861 10.1182/blood-2014-05-378638 PMC4148764

[17] Sadler JE . Biochemistry and genetics of von Willebrand factor. Annu Rev Biochem. 1998;67:395‐424. 9759493 10.1146/annurev.biochem.67.1.395

[18] Wang JW , Valentijn KM , de Boer HC , et al. Intracellular storage and regulated secretion of von Willebrand factor in quantitative von Willebrand disease. J Biol Chem. 2011;286(27):24180‐24188. 21596755 10.1074/jbc.M110.215194 PMC3129199

[19] Randi AM , Laffan MA . Von Willebrand factor and angiogenesis: basic and applied issues. J Thromb Haemost. 2017;15(1):13‐20. 27778439 10.1111/jth.13551

[20] Metcalf DJ , Nightingale TD , Zenner HL , Lui‐Roberts WW , Cutler DF . Formation and function of Weibel‐Palade bodies. J Cell Sci. 2008;121(Pt 1):19‐27. 18096688 10.1242/jcs.03494

[21] Voorberg J , Fontijn R , Calafat J , Janssen H , van Mourik JA , Pannekoek H . Biogenesis of von Willebrand factor‐containing organelles in heterologous transfected CV‐1 cells. EMBO J. 1993;12(2):749‐758. 8440262 10.1002/j.1460-2075.1993.tb05709.x PMC413262

[22] Wagner DD , Saffaripour S , Bonfanti R , et al. Induction of specific storage organelles by von Willebrand factor propolypeptide. Cell. 1991;64(2):403‐413. 1988154 10.1016/0092-8674(91)90648-i

[23] Zhou Y‐F , Eng ET , Nishida N , Lu C , Walz T , Springer TA . A pH‐regulated dimeric bouquet in the structure of von Willebrand factor. EMBO J. 2011;30(19):4098‐4111. 21857647 10.1038/emboj.2011.297 PMC3209782

[24] Doddaballapur A , Michalik Katharina M , Manavski Y , et al. Laminar shear stress inhibits endothelial cell metabolism via KLF2‐mediated repression of PFKFB3. Arterioscler Thromb Vasc Biol. 2015;35(1):137‐145. 25359860 10.1161/ATVBAHA.114.304277

[25] Rondaij MG , Bierings R , Kragt A , van Mourik JA , Voorberg J . Dynamics and plasticity of Weibel‐Palade bodies in endothelial cells. Arterioscler Thromb Vasc Biol. 2006;26(5):1002‐1007. 16469951 10.1161/01.ATV.0000209501.56852.6c

[26] Chistiakov DA , Orekhov AN , Bobryshev YV . Effects of shear stress on endothelial cells: go with the flow. Acta Phys. 2017;219(2):382‐408. 10.1111/apha.12725 27246807

[27] Potente M , Gerhardt H , Carmeliet P . Basic and therapeutic aspects of angiogenesis. Cell. 2011;146(6):873‐887. 21925313 10.1016/j.cell.2011.08.039

[28] Harrison‐Lavoie KJ , Michaux G , Hewlett L , et al. P‐Selectin and CD63 use different mechanisms for delivery to Weibel‐Palade bodies. Traffic. 2006;7(6):647‐662. 16683915 10.1111/j.1600-0854.2006.00415.x

[29] Dekker RJ , Boon RA , Rondaij MG , et al. KLF2 provokes a gene expression pattern that establishes functional quiescent differentiation of the endothelium. Blood. 2006;107(11):4354‐4363. 16455954 10.1182/blood-2005-08-3465

[30] Wang G , Kostidis S , Tiemeier GL , et al. Shear stress regulation of endothelial glycocalyx structure is determined by glucobiosynthesis. Arterioscler Thromb Vasc Biol. 2019;40(2):350‐364. 31826652 10.1161/ATVBAHA.119.313399

[31] Enge M , Bjarnegard M , Gerhardt H , et al. Endothelium‐specific platelet‐derived growth factor‐B ablation mimics diabetic retinopathy. EMBO J. 2002;21(16):4307‐4316. 12169633 10.1093/emboj/cdf418 PMC126162

[32] Ferland‐McCollough D , Slater S , Richard J , Reni C , Mangialardi G . Pericytes, an overlooked player in vascular pathobiology. Pharmacol Ther. 2017;171:30‐42. 27916653 10.1016/j.pharmthera.2016.11.008 PMC6008604

[33] Yoshioka N , Gros E , Li HR , Kumar S , Deacon DC , Maron C , Muotri AR , Chi NC , Fu XD , Yu BD , et al. Efficient generation of human iPSCs by a syntheticself‐replicative RNA. Cell stem cell. 2013;13:246‐254. 23910086 10.1016/j.stem.2013.06.001 PMC3845961

[34] Gesa L . Tiemeier GW , Sébastien JD . Wendy M.P.J.S. , M.C. Avramut , T. Karakach , V. V. Orlova , C.W. van den Berg , C.L. Mummery , P. Carmeliet , B.M. van den Berg , T.J. Rabelink . Closing the mitochondrial permeability transition pore in hiPSC‐derived endothelial cells induces Glyococalyx formation and functional maturation. Stem Cell Rep. 2019;Vol.1 13:1–14. 10.1016/j.stemcr.2019.10.005 PMC6895683 31680061

[35] Leuning DG , Reinders ME , Li J , et al. Clinical‐grade isolated human kidney perivascular stromal cells as an Organotypic cell source for kidney regenerative medicine. Stem Cells Trans Med. 2017;6(2):405‐418. 10.5966/sctm.2016-0053 PMC5442810 28191776

[36] Nguyen U , Squaglia N , Boge A , Fung PA . The simple Western™: a gel‐free, blot‐free, hands‐free Western blotting reinvention. Nat Methods. 2011;8:982.

[37] Dobin A , Davis CA , Schlesinger F , et al. STAR: ultrafast universal RNA‐seq aligner. Bioinformatics. 2013;29(1):15‐21. 23104886 10.1093/bioinformatics/bts635 PMC3530905

[38] Li B , Dewey CN . RSEM: accurate transcript quantification from RNA‐Seq data with or without a reference genome. BMC Bioinformatics. 2011;12:323. 21816040 10.1186/1471-2105-12-323 PMC3163565

[39] de Jong A , Dirven RJ , Oud JA , Tio D , van Vlijmen BJM , Eikenboom J . Correction of a dominant‐negative von Willebrand factor multimerization defect by small interfering RNA‐mediated allele‐specific inhibition of mutant von Willebrand factor. J Thromb Haemost. 2018;16(7):1357‐1368. 29734512 10.1111/jth.14140

[40] van Borren MM , den Ruijter HM , Baartscheer A , Ravesloot JH , Coronel R , Verkerk AO . Dietary omega‐3 polyunsaturated fatty acids suppress NHE‐1 upregulation in a rabbit model of volume‐ and pressure‐overload. Front Physiol. 2012;3:76. 22485092 10.3389/fphys.2012.00076 PMC3317268

[41] Kostidis S , Addie RD , Morreau H , Mayboroda OA , Giera M . Quantitative NMR analysis of intra‐ and extracellular metabolism of mammalian cells: a tutorial. Anal Chim Acta. 2017;980:1‐24. 28622799 10.1016/j.aca.2017.05.011

[42] Vinaixa M , Rodríguez MA , Aivio S , et al. Positional enrichment by proton analysis (PEPA): a one‐dimensional 1H‐NMR approach for 13C stable isotope tracer studies in metabolomics. Angew Chem Int Ed. 2017;56(13):3531‐3535. 10.1002/anie.201611347 PMC5363230 28220994

[43] Gu M , Shao NY , Sa S , et al Patient‐specific iPSC‐derived endothelial cells uncover pathways that protect against pulmonary hypertension in BMPR2 mutation carriers. Cell Stem Cell. 2017;20(4):490‐504.e495. 28017794 10.1016/j.stem.2016.08.019 PMC5500296

[44] Feliciangeli SF , Thomas L , Scott GK , et al. Identification of a pH sensor in the furin propeptide that regulates enzyme activation. J Biol Chem. 2006;281(23):16108‐16116. 16601116 10.1074/jbc.M600760200 PMC4293020

[45] Wagner DD , Mayadas T , Marder VJ . Initial glycosylation and acidic pH in the Golgi apparatus are required for multimerization of von Willebrand factor. J Cell Biol. 1986;102(4):1320‐1324. 3082891 10.1083/jcb.102.4.1320 PMC2114173

[46] Llopis J , McCaffery JM , Miyawaki A , Farquhar MG , Tsien RY . Measurement of cytosolic, mitochondrial, and Golgi pH in single living cells with green fluorescent proteins. Proc Natl Acad Sci USA. 1998;95(12):6803‐6808. 9618493 10.1073/pnas.95.12.6803 PMC22642

[47] Casey JR , Grinstein S , Orlowski J . Sensors and regulators of intracellular pH. Nat Rev Mol Cell Biol. 2010;11(1):50‐61. 19997129 10.1038/nrm2820

[48] Crimi E , Taccone FS , Infante T , Scolletta S , Crudele V , Napoli C . Effects of intracellular acidosis on endothelial function: an overview. J Crit Care. 2012;27(2):108‐118. 21798701 10.1016/j.jcrc.2011.06.001

[49] Von GV . Willebrand factor folds into a bouquet. EMBO J. 2011;30(19):3880‐3881. 21975375 10.1038/emboj.2011.321 PMC3209786

[50] Mollenhauer HH , Morre DJ , Rowe LD . Alteration of intracellular traffic by monensin; mechanism, specificity and relationship to toxicity. Biochim Biophys Acta. 1990;1031(2):225‐246. 2160275 10.1016/0304-4157(90)90008-Z PMC7148783

[51] De Bock K , Georgiadou M , Schoors S , et al. Role of PFKFB3‐driven glycolysis in vessel sprouting. Cell. 2013;154(3):651‐663. 23911327 10.1016/j.cell.2013.06.037

[52] Eelen G , de Zeeuw P , Treps L , Harjes U , Wong BW , Carmeliet P . Endothelial cell metabolism. Physiol Rev. 2018;98(1):3‐58. 29167330 10.1152/physrev.00001.2017 PMC5866357

[53] Krutzfeldt A , Spahr R , Mertens S , Siegmund B , Piper HM . Metabolism of exogenous substrates by coronary endothelial cells in culture. J Mol Cell Cardiol. 1990;22(12):1393‐1404. 2089157 10.1016/0022-2828(90)90984-a

[54] Delvin TM . Textbook of Biochemistry with Clinical Correlations. 6th ed. New York, NY: Wiley‐Liss; 2006.

[55] Berg JLT JM, Tryer L, Gatto GJ Jr, eds. Biochemistry. 7th ed. W.H. New York, NY: Freeman and Company; 2012.

[56] Dyck JR , Lopaschuk GD . Glucose metabolism, H+ production and Na+/H+‐exchanger mRNA levels in ischemic hearts from diabetic rats. Mol Cell Biochem. 1998;180(1–2):85‐93. 9546634

[57] Cliff TS , Wu T , Boward BR , et al MYC controls human pluripotent stem cell fate decisions through regulation of metabolic flux. Cell Stem Cell. 2017;21(4):502‐516.e509. 10.1016/j.stem.2017.08.018 PMC5644510 28965765

[58] Sonveaux P , Copetti T , De Saedeleer CJ , et al. Targeting the lactate transporter MCT1 in endothelial cells inhibits lactate‐induced HIF‐1 activation and tumor angiogenesis. PLoS One. 2012;7(3):e33418. 22428047 10.1371/journal.pone.0033418 PMC3302812

[59] Halestrap AP , Wilson MC . The monocarboxylate transporter family—role and regulation. IUBMB Life. 2012;64(2):109‐119. 22162139 10.1002/iub.572

[60] Uhernik AL , Tucker C , Smith JP . Control of MCT1 function in cerebrovascular endothelial cells by intracellular pH. Brain Res. 2011;1376:10‐22. 21192921 10.1016/j.brainres.2010.12.060

[61] Rafii S , Butler JM , Ding B‐S . Angiocrine functions of organ‐specific endothelial cells. Nature. 2016;529(7586):316‐325. 26791722 10.1038/nature17040 PMC4878406

[62] Sun S , Li H , Chen J , Qian Q . Lactic acid: no longer an inert and end‐product of glycolysis. Physiology. 2017;32(6):453‐463. 29021365 10.1152/physiol.00016.2017

[63] Hui S , Ghergurovich JM , Morscher RJ , et al. Glucose feeds the TCA cycle via circulating lactate. Nature. 2017;551:115‐118. 29045397 10.1038/nature24057 PMC5898814

[64] Goehring NW , Hyman AA . Organelle growth control through limiting pools of cytoplasmic components. Curr Biol. 2012;22(9):R330‐R339. 22575475 10.1016/j.cub.2012.03.046

[65] Ferraro F , Kriston‐Vizi J , Metcalf Daniel J , et al. A two‐tier Golgi‐based control of organelle size underpins the functional plasticity of endothelial cells. Dev Cell. 2014;29(3):292‐304. 24794632 10.1016/j.devcel.2014.03.021 PMC4022834

[66] McCormack JJ , Harrison‐Lavoie K , Cutler DF . Human Endothelial Cells Size‐Select their Secretory Granules for Exocytosis to Modulate their Functional Output. JTH: J Thromb Haemost; 2019. 10.1111/jth.14634 PMC7155122 31519030

[67] Maisonpierre PC , Suri C , Jones PF , et al. Angiopoietin‐2, a natural antagonist for Tie2 that disrupts in vivo angiogenesis. Science. 1997;277(5322):55‐60. 9204896 10.1126/science.277.5322.55

[68] Zhang L , Yang N , Park JW , et al. Tumor‐derived vascular endothelial growth factor up‐regulates angiopoietin‐2 in host endothelium and destabilizes host vasculature, supporting angiogenesis in ovarian cancer. Cancer Res. 2003;63(12):3403‐3412. 12810677

[69] van den Berg CW , Ritsma L , Avramut MC , et al. Renal subcapsular transplantation of PSC‐derived kidney Organoids induces neo‐vasculogenesis and significant glomerular and tubular maturation in vivo. Stem Cell Rep. 2018;10(3):751‐765. 10.1016/j.stemcr.2018.01.041 PMC5918682 29503086

