# Supplementary material for: Lowering the increased intracellular pH of human‐induced pluripotent stem cell‐derived endothelial cells induces formation of mature Weibel‐Palade bodies
Source: Stem Cells Transl Med. 2020 Mar 12;9(7):758–72. doi: 10.1002/sctm.19-0392 (PMC7308639; doi:10.1002/sctm.19-0392)
Supplement: Supplementary file 1 — Appendix S1: Supplemental data Figure S1. Comparison of hiPSC‐EC differentiation protocols by RNA‐sequencing Figure S2. Lack of Weibel‐Palade bodies in hiPSC‐ECs Figure S3. hiPSC‐ECs WPB phenotype of KLF2 transduced cells Figure S4. Effects of acetic acid addition in hiPSC‐ECs [file SCT3-9-758-s001.pdf]

Supplemental Information

Supplemental data

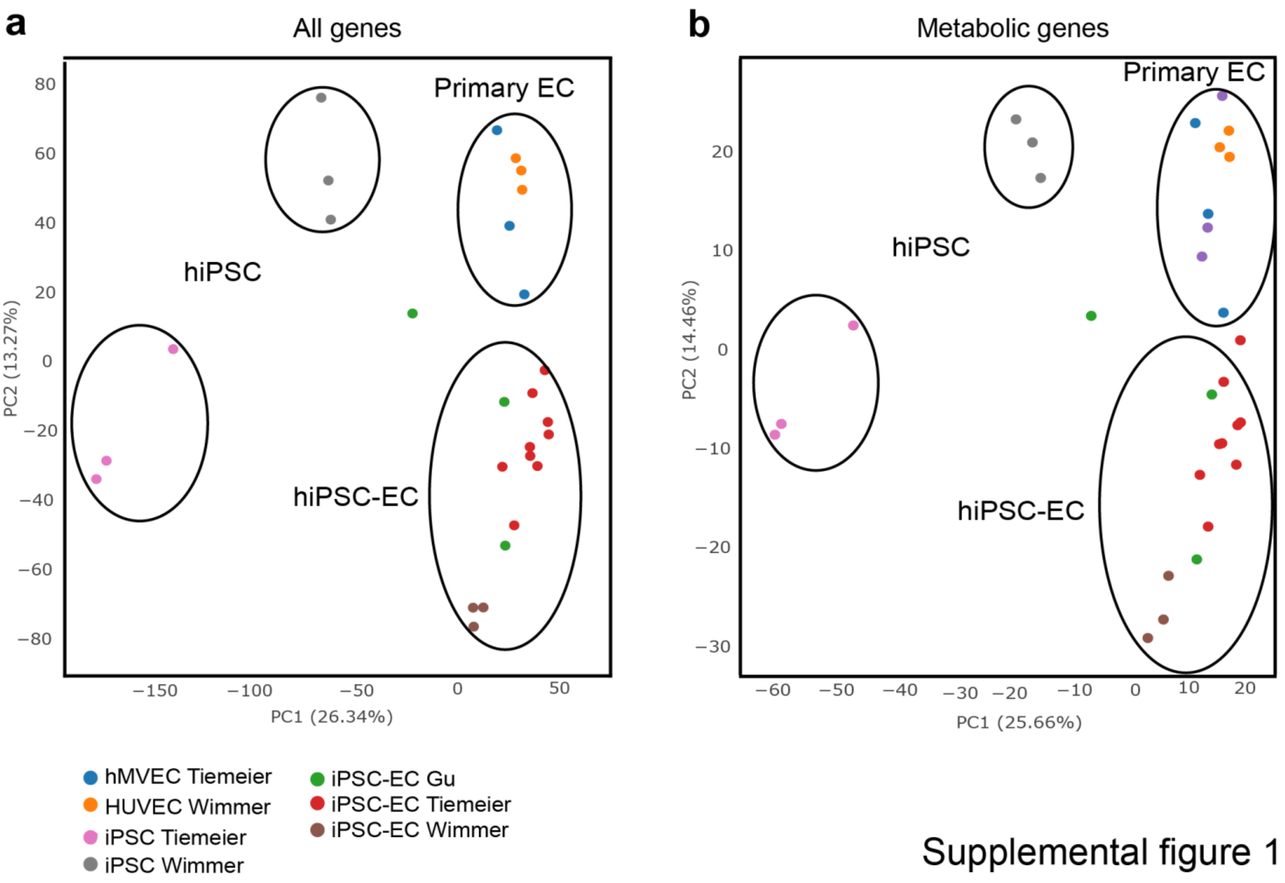

Supplemental figure 1 **Comparison of hiPSC-EC differentiation protocols by RNA-sequencing**

Principle component analysis of all genes **(a)** and metabolic genes **(b)** acquired from RNA-sequencing results of hiPSC (Wimmer et al: pink , Tiemeier et al: grey), primary mature ECs (HUVEC: orange, hMVECs: blue) and hiPSC-ECs derived by several differentiation protocols (Wimmer et al: brown, Gu et al: green, Tiemeier et al: red) in static 2D conditions. Tiemeier et al. used three hiPSC lines to create hiPSC-ECs: NCRM1, L72, L99.

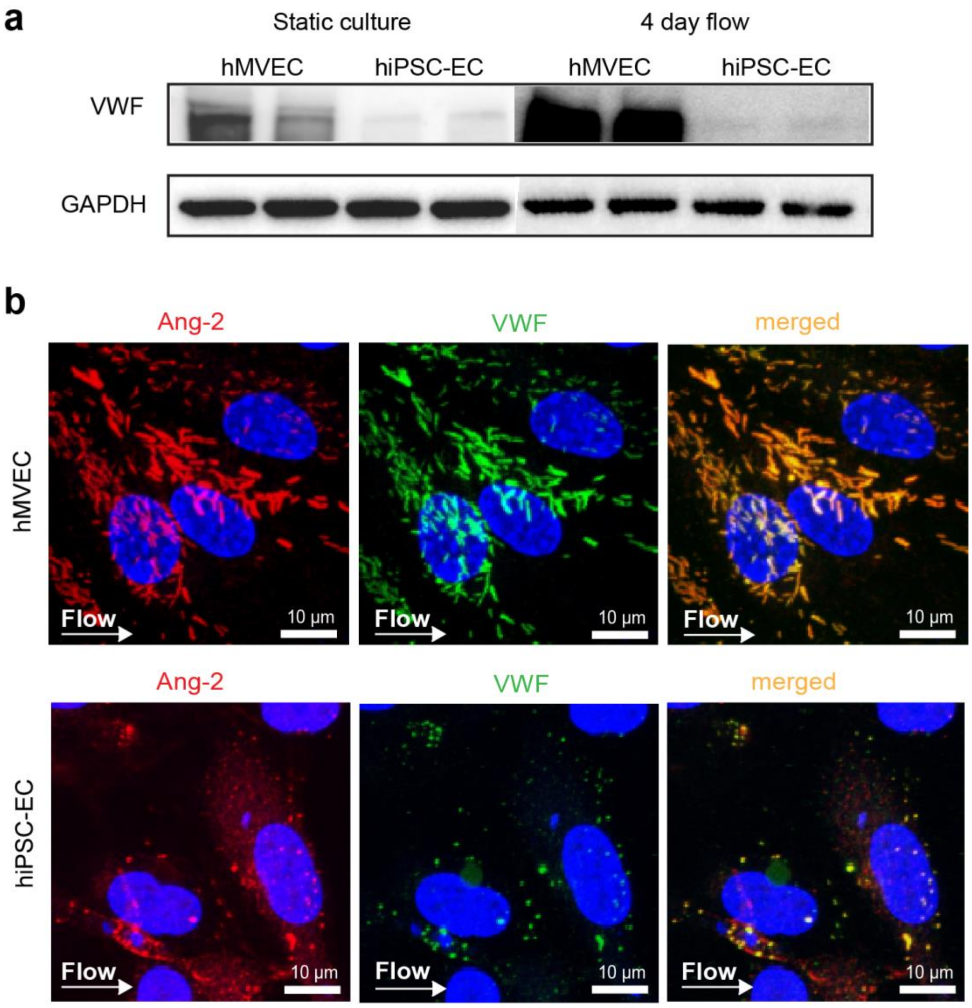

Supplemental figure 2

Supplemental figure 2 **Lack of Weibel Palade Bodies in hiPSC-ECs.**

(a) Western blot analyses of VWF protein expression of hMVECs and hiPSC-ECs NCRM1 after static cell culture and after 4 days laminar flow. GAPDH was included as a positive control. Blots are representative of three independent experiments. **(b)** Representative cross-sectional confocal images stained for VWF (green) and Ang-2 (red) of hMVECs and hiPSC-ECs NCRM1.

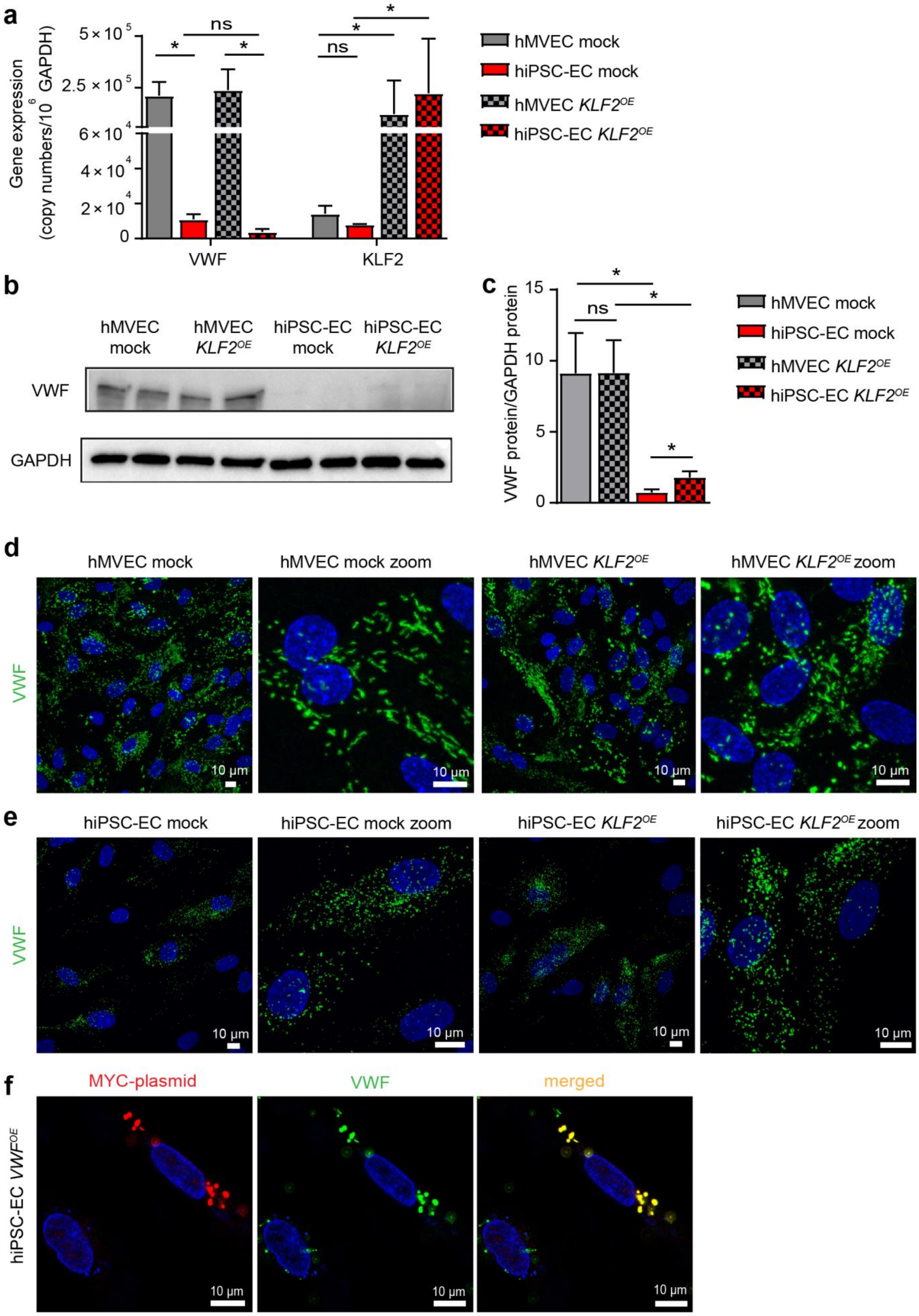

Supplemental figure 3

Supplemental figure 3 **hiPSC-ECs WPB phenotype of KLF2 transduced cells.**

(a) qPCR results of KLF2 and VWF expression in hMVECs and hiPSC-ECs NCRM1 mock and lentiviral KLF2 transduced cells (*KLF2*^{OE}). **(b)** Western blot analyses of VWF protein expression in hMVECs and hiPSC-ECs NCRM1 mock and after KLF2 transduction. GAPDH was included as a positive control. Blots are representative of three independent experiments. **(c)** Quantification of western blot analyses of VWF protein expression in hMVECs and hiPSC-ECs NCRM1 mock and *KLF2*^{OE} of hMVECs and hiPSC-ECs NCRM1. GAPDH was included as a positive control. **(d)** Representative cross-sectional confocal images stained for VWF (green) of hMVECs mock and *KLF2*^{OE}. **(e)** Representative cross-sectional confocal images stained for VWF (green) of hiPSC-ECs NCRM1 mock and *KLF2*^{OE}. **(g)** Representative cross-sectional confocal images stained for VWF (green) and MYC-plasmid (red) of hiPSC-ECs NCRM1 after VWF transfection (*VWF*^{OE}).

Values are given as mean ± SEM of 3 independent experiments. One-way ANOVA was performed; *P < 0.05, **P < 0.001, ***P < 0.0001.

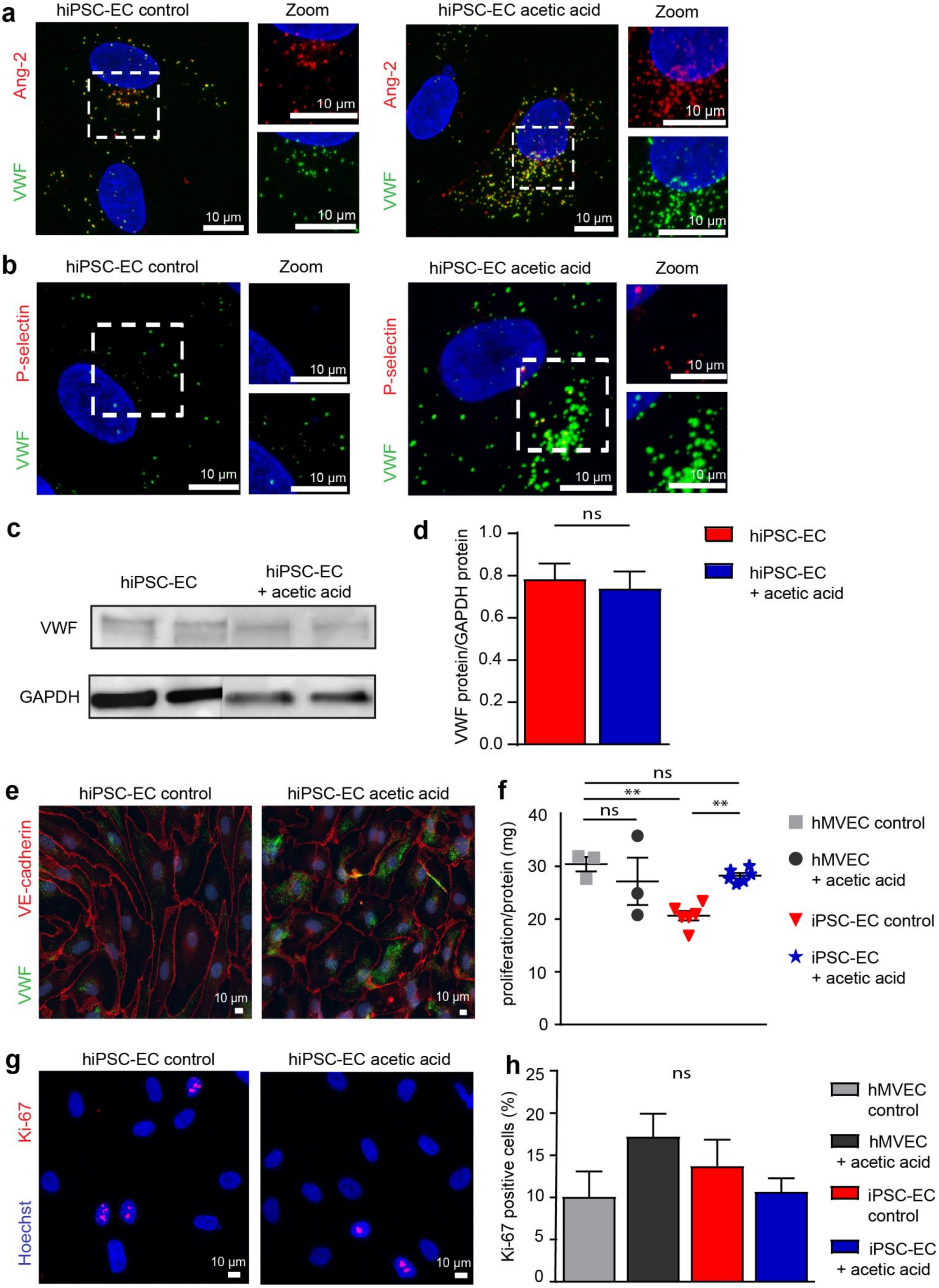

Supplemental figure 4

Supplemental figure 4 **Effects of acetic acid addition in hiPSC-ECs**

(a) Representative cross-sectional confocal images of iPSC-ECs stained for Ang-2 (red), VWF (green) and Hoechst (blue) after 24h of culture with 10mM acetic acid.

(b) Representative cross-sectional confocal images of iPSC-ECs stained for P-selectin (red), VWF (green) and Hoechst (blue) after 24h of culture with 10mM acetic acid. **(c)** Western blot analyses of VWF protein expression in hiPSC-ECs NCRM1 control and after culturing in acetate acid (10 mM) containing medium. GAPDH was included as a positive control. Both taken from same blot. Blots are representative of three independent experiments. **(d)** Quantification of western blot analyses of VWF protein expression in hiPSC-ECs NCRM1 control and of hiPSC-ECs NCRM1 cultured in acetate acid (10 mM) containing medium. GAPDH was included as a positive control. **(e)** Representative cross-sectional confocal images of iPSC-ECs stained for VE-cadherin (red), VWF (green) and Hoechst (blue) after 24h of culture with 10mM acetic acid. **(f)** Proliferation rate of hMVEC and hiPSC-ECs measured by MTT assay normalized by mg protein after 24h culture with 10mM of acetic acid. **(g)** Representative cross-sectional confocal images of iPSC-ECs stained for ki-67 (red) and Hoechst (blue) after 24h of culture with 10mM acetic acid. **(h)** Quantification of ki-67 positive cells (n=200-500).

Values are given as mean ± SEM of 3 independent experiments. Non-paired 2-tailed Student's t-test or One-Way ANOVA were performed; *P < 0.05, **P < 0.001, ***P < 0.0001, ns = non-significant.
